# The rapid *in vivo* evolution of *Pseudomonas aeruginosa* in ventilator-associated pneumonia patients leads to attenuated virulence

**DOI:** 10.1098/rsob.170029

**Published:** 2017-09-06

**Authors:** Ke Wang, Yi-qiang Chen, May M. Salido, Gurjeet S. Kohli, Jin-liang Kong, Hong-jie Liang, Zi-ting Yao, Yan-tong Xie, Hua-yu Wu, Shuang-qi Cai, Daniela I. Drautz-Moses, Aaron E. Darling, Stephan C. Schuster, Liang Yang, Yichen Ding

**Affiliations:** 1 Department of Respiratory Disease, First Affiliated Hospital of Guangxi Medical University, Nanning 530021, Guangxi, People's Republic of China; 2 Department of Clinical Laboratory, First Affiliated Hospital of Guangxi Medical University, Nanning 530021, Guangxi, People's Republic of China; 3 Centre for Genomic and Personalized Medicine, Guangxi Medical University, Nanning 530021, Guangxi, People's Republic of China; 4 Department of Cell Biology and Genetics, Guangxi Medical University, Nanning 530021, Guangxi, People's Republic of China; 5 Singapore Centre for Environmental Life Sciences Engineering (SCELSE), Nanyang Technological University, Singapore 637551, Singapore; 6 School of Biological Sciences, Nanyang Technological University, Singapore 637551, Singapore; 7 The First Clinical School of Guangxi Medical University, Nanning 530021, Guangxi, People's Republic of China; 8 The ithree Institute, University of Technology Sydney, Sydney, New South Wales, Australia; 9 Interdisciplinary Graduate School, SCELSE, Nanyang Technological University, Singapore 639798, Singapore

**Keywords:** *Pseudomonas aeruginosa*, ventilator-associated pneumonia, *in vivo* evolution, genomics, adaptation

## Abstract

*Pseudomonas aeruginosa* is an opportunistic pathogen that causes severe airway infections in humans. These infections are usually difficult to treat and associated with high mortality rates. While colonizing the human airways, *P. aeruginosa* could accumulate genetic mutations that often lead to its better adaptability to the host environment. Understanding these evolutionary traits may provide important clues for the development of effective therapies to treat *P. aeruginosa* infections. In this study, 25 *P. aeruginosa* isolates were longitudinally sampled from the airways of four ventilator-associated pneumonia (VAP) patients. Pacbio and Illumina sequencing were used to analyse the *in vivo* evolutionary trajectories of these isolates. Our analysis showed that positive selection dominantly shaped *P. aeruginosa* genomes during VAP infections and led to three convergent evolution events, including loss-of-function mutations of *lasR* and *mpl*, and a pyoverdine-deficient phenotype. Specifically, *lasR* encodes one of the major transcriptional regulators in quorum sensing, whereas *mpl* encodes an enzyme responsible for recycling cell wall peptidoglycan. We also found that *P. aeruginosa* isolated at late stages of VAP infections produce less elastase and are less virulent *in vivo* than their earlier isolated counterparts, suggesting the short-term *in vivo* evolution of *P. aeruginosa* leads to attenuated virulence.

## 1. Introduction

The opportunistic pathogen *Pseudomonas aeruginosa* is one of the leading causes of nosocomial infections worldwide [1]. It was estimated that the average cost for each *P. aeruginosa* infection case is US $24 700 with a median hospitalization period of 45 days [2]. Among the various types of infections caused by *P. aeruginosa*, lung infections have become a major concern. *Pseudomonas aeruginosa* was reported to be the most frequently isolated bacteria from the respiratory tract and one of the major causes of ventilator-associated pneumonia (VAP) [3]. In addition, *P. aeruginosa* is also the major pathogen causing lung infections and death in patients suffering from cystic fibrosis (CF) and chronic obstructive pulmonary disease [4].

*Pseudomonas aeruginosa* harbours multiple virulence factors that have been proved to be essential for causing lung infections. For instance, *P. aeruginosa* quorum-sensing systems and regulated virulence factors such as rhamnolipids and elastase were shown to play important roles in its lung colonization [5,6]. The formation of surface-attached *P. aeruginosa* biofilm communities was implicated in persistent lung infections of VAP patients and CF patients [7,8], which raises a great challenge for the development of effective antimicrobial therapy.

Previous studies have shown that *P. aeruginosa* can undergo adaptive evolution during lung infections. Understanding these evolutionary traits may help to predict novel targets for the design of effective treatment strategies [9]. For instance, both *lasR* and pyoverdine-deficient *P. aeruginosa* mutants have been identified in the CF lungs [9,10]. LasR is a transcriptional regulator that can activate the expression of various virulence factors in response to *N*-(3-oxododecanoyl)-l-homoserine lactone signal, whereas pyoverdine is a siderophore by which *P. aeruginosa* scavenges ferric iron from the environment and within the host [11,12]. The accumulation of these mutants in the CF lungs suggests that remodelling of the quorum-sensing regulatory system and iron metabolism is crucial for the adaptation of *P. aeruginosa* to the host environment. In addition, *lasR* mutants were also reported to evolve from the wild-type in mechanically ventilated patients within 10 days of colonization [13]. The evolution of *lasR* mutants is probably because they could gain fitness advantages by exploiting exoproducts of the wild-type [13]. Although these studies have provided insights into the adaptive evolution of *P. aeruginosa* during chronic and acute pulmonary infections, there is little evidence showing the evolutionary dynamics of *P. aeruginosa* during acute pulmonary infections. To address this, we investigated the adaptive evolution of *P. aeruginosa* during acute VAP infections by sequencing the genomes of 25 *P. aeruginosa* isolates sequentially sampled from four VAP patients. We show that the short-term *in vivo* evolution of *P. aeruginosa* in VAP patients leads to attenuated virulence.

## 2. Results

### 2.1. A novel *Pseudomonas aeruginosa* strain causing nosocomial outbreak of ventilator-associated pneumonia

Four patients developed VAP infections after admission into the intensive care units of a teaching hospital in China from December 2013 to March 2014. Cultivation of the patients' sputum samples and 16s rDNA PCR suggested that *P. aeruginosa* was responsible for their VAP infections. In the initial screening, we randomly selected three colonies cultured from each sputum sample and compared their colony morphologies, antibiotic resistance and pigment production. We found that the three colonies isolated from the same sputum sample showed no phenotypic variations to each other. Therefore, to investigate the epidemic linkage and adaptive evolution of *P. aeruginosa* during VAP infections, one single colony from each sputum sample was sequenced to represent the dominant clone. Patient information and the sampling time for each isolate can be found in the electronic supplementary material, table S1.

The genomes of 24 isolates (PA_D1 to PA_D24) were sequenced on an Illumina HiSeq 2500 platform and one isolate (PA_D25) was sequenced on an Illumina MiSeq platform. We further sequenced the earliest and latest isolate from each patient on a Pacific Biosciences RSII sequencer to obtain their concise genome maps. Illumina and Pacific Biosciences sequencing achieved on-average 100× and 50× coverage of the *P. aeruginosa* genomes, respectively. These sequencing reads were successfully assembled into eight circular genomes and 17 draft genomes. The general characteristics of the eight fully sequenced genomes can be found in the electronic supplementary material, table S2.

**Figure 1.**
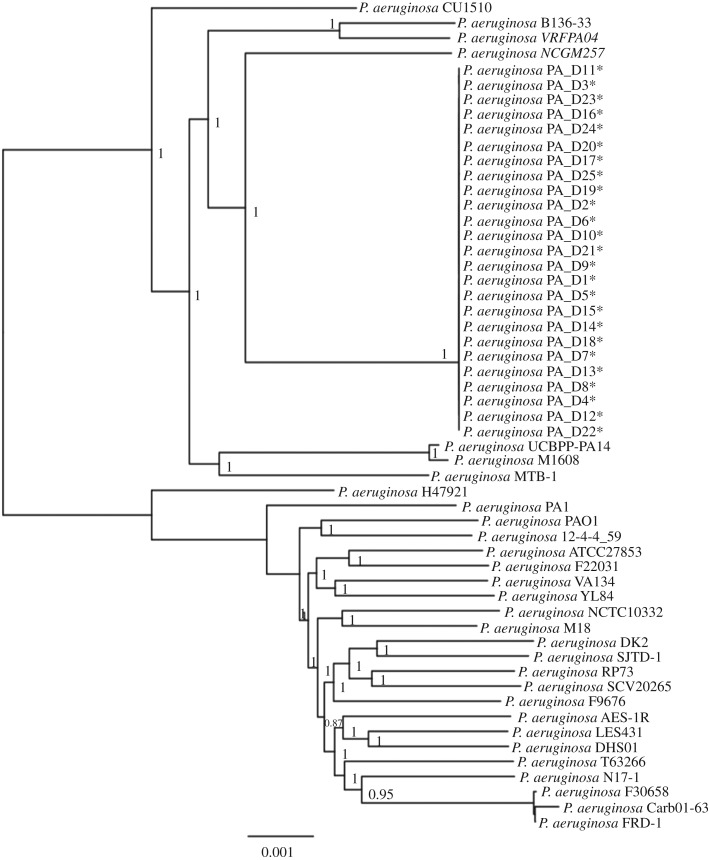
Phylogenetic analysis of the 25 sequenced *P. aeruginosa* genomes and 30 other *P. aeruginosa* genomes. The 25 *P. aeruginosa* isolates sequenced in this study cluster together and form a monophyletic group (asterisk indicates isolate sequenced in this study). Phylogenetic inference was then carried out based on 132 511 variant sites using the approximate maximum-likelihood algorithm, with clade confidence estimated with SH-like support values. Accession numbers of the 30 genomes from GenBank are listed in the electronic supplementary material, table S3.

To identify the origin of the 25 isolates in our study, we analysed the phylogeny of the 25 genomes against 30 other *P. aeruginosa* genomes from GenBank. The phylogenetic tree based on core-genome alignment showed that the 25 genomes are closely related to each other and form a monophyletic group (figure 1). In addition, we performed random amplified polymorphic DNA (RAPD) typing on the 25 isolates. The results suggested that the 25 isolates are of the same clone (electronic supplementary material, figure S1).

### 2.2. General characteristics of *Pseudomonas aeruginosa* PA_D1 genome

As the genomes of the 25 isolates are closely related (figure 1; electronic supplementary material, figure S1), we focused on characterizing PA_D1 genome as a representative strain of this study. The PA_D1 genome consists of a circular chromosome comprising 6 643 823 bp with an average GC content of 66.2%. Annotation on Rapid Annotations using Subsystem Technology (RAST) Server [14] showed that PA_D1 genome is predicted to encode 6210 genes, of which 6135 are protein-encoding genes and 75 are RNA-coding genes (63 tRNAs, 12 rRNAs; electronic supplementary material, table S2). Among the 6135 protein-encoding genes, 4918 can be assigned to putative functions, with the remaining annotated as hypothetical proteins.

**Figure 2.**
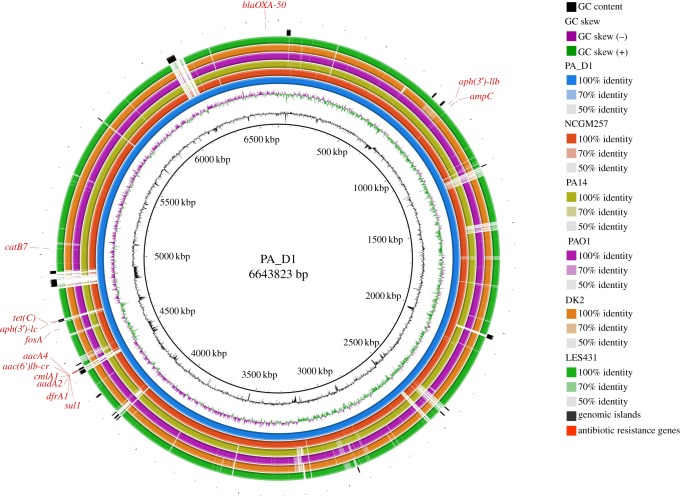
Sequence conservation between PA_D1 and five other *P. aeruginosa* genomes. From the innermost to outermost: circle 1, PA_D1, the representative strain in this study; circle 2, NCGM257, a *P. aeruginosa* strain that causes nosocomial infections in Japan, which is also the closest genome to isolates in this study as shown by the phylogenetic analysis; circle 3, PA14, a highly virulent strain; circle 4, PAO1, the laboratory reference strain; circle 5, DK2, a strain isolated from CF lungs; circle 6, LES431, an endemic strain isolated in UK; circle 7, GIs predicted by IslandViewer 3 [15]; circle 8, antibiotic resistance genes predicted by ResFinder 2.1 server [16].

Alignment of the PA_D1 genome with five other *P. aeruginosa* genomes revealed that it harbours several strain-specific genomic regions (figure 2). To identify the cause of these strain-specific genomic regions, we predicted the genomic islands (GIs) present in the PA_D1 genome using IslandViewer 3 server [17]. The 31 predicted GIs in the PA_D1 genome correlate well with its strain-specific regions (figure 2; electronic supplementary material, table S4), suggesting that most of the strain-specific regions can be explained by horizontal gene transfer events. Notably, many of the genes present in the GIs encode products involved in generating transposons, virulence, energy metabolisms, antibiotic resistance and DNA methylation and repair (electronic supplementary material, table S4), suggesting that these GIs are important for PA_D1 to survive under stressful conditions and subsequent spreading in nosocomial settings.

### 2.3. Acquisition of antibiotic resistance

To identify the antibiotic resistance profiles of the 25 isolates, we performed antibiotic susceptibility tests. The results showed that all the isolates displayed resistance to various classes of antibiotics, including macrolides (azithromycin), penicillins and penicillin combined with beta-lactamase inhibitor (ampicillin, piperacillin-tazobactam), cephalosporins (cefazolin, cefotetan, ceftazidime, ceftriaxone, cefepime), aminoglycosides (gentamicin, tobramycin), fluoroquinolones (ciprofloxacin, levofloxacin), but remained sensitive to amikacin (table 1). Interestingly, 19 isolates from patient 1, patient 2 and patient 3 remained sensitive to imipenem, whereas the six isolates from patient 4 showed resistance to imipenem, which belongs to carbapenem class of antibiotics. Prediction of resistance gene on ResFinder 2.1 server [16] showed that all the 25 isolates harbour the same set of antibiotic resistance genes. These genes are responsible for resistance to aminoglycosides (*aadA2*, *aacA4*, *aph(3')-Ic* and *aph(3')-IIb*), beta-lactams (*bla_OXA−50_* and *ampC*), fluoroquinolones (*aac(6')Ib-cr*), fosfomycins (*fosA*), phenicols (*cmlA1* and *catB7*), sulfonamide (*sul1*), trimethoprim (*dfrA1*) and tetracycline (*tet(C)*). Intriguingly, eight out of the 13 predicted antibiotic resistance genes are located in the predicted GIs (figure 2), suggesting that horizontal gene transfer is responsible for the acquisition of antibiotic resistance genes in these isolates.

**Table 1.** Antibiotic susceptibility profiles of 25 *P. aeruginosa* isolates. AMK, amikacin; AMP, ampicillin; ATM, azithromycin; CAZ, ceftazidime; CIP, ciprofloxacin; CRO, ceftriaxone; CTT, cefotetan; CZO, cefazolin; FEP, cefepime; GEM, gentamicin; IMP, imipenem; LVX, levofloxacin; TOB, Tobramycin; TZP, piperacillin-tazobactam. (unit: mg l^−1^).

	AMK	AMP	ATM	CAZ	CIP	CRO	CTT	CZO	FEP	LVX	GEN	IPM	TOB	TZP
*Patient 1*														
PA_D1	4	≥32	16	4	≥4	≥64	≥64	≥64	8	≥8	≥16	≤1	≥16	16
PA_D3	≤2	≥32	16	16	≥4	≥64	≥64	≥64	8	≥8	≥16	≤1	≥16	64
PA_D8	≤2	≥32	16	16	≥4	≥64	≥64	≥64	8	≥8	≥16	≤1	≥16	64
PA_D9	≤2	≥32	16	16	≥4	≥64	≥64	≥64	4	≥8	≥16	2	≥16	32
*Patient 2*														
PA_D2	4	≥32	16	16	≥4	≥64	≥64	≥64	8	≥8	≥16	≤1	≥16	64
PA_D4	16	≥32	16	4	≥4	≥64	≥64	≥64	8	≥8	≥16	≤1	≥16	64
PA_D6	≤2	≥32	16	8	≥4	≥64	≥64	≥64	8	≥8	≥16	≤1	≥16	64
PA_D10	≤2	≥32	≥64	16	≥4	≥64	≥64	≥64	8	≥8	≥16	≤1	≥16	64
PA_D12	≤2	≥32	≥64	≥64	≥4	≥64	≥64	≥64	16	≥8	≥16	2	≥16	≥128
PA_D19	≤2	≥32	32	4	≥4	≥64	≥64	≥64	8	≥8	≥16	2	≥16	64
PA_D21	4	≥32	32	32	≥4	≥64	≥64	≥64	8	≥8	≥16	≤1	≥16	64
*Patient 3*														
PA_D5	≤2	≥32	16	4	≥4	≥64	≥64	≥64	4	≥8	≥16	≤1	≥16	16
PA_D7	≤2	≥32	16	16	≥4	≥64	≥64	≥64	8	≥8	≥16	≤1	≥16	64
PA_D11	≤2	≥32	16	8	≥4	≥64	≥64	≥64	4	≥8	≥16	2	≥16	64
PA_D13	≤2	≥32	16	16	≥4	≥64	≥64	≥64	8	≥8	≥16	2	≥16	64
PA_D14	≤2	≥32	16	16	≥4	≥64	≥64	≥64	8	≥8	≥16	2	≥16	64
PA_D15	≤2	≥32	16	16	≥4	≥64	≥64	≥64	8	≥8	≥16	≤1	≥16	64
PA_D18	≤2	≥32	16	16	≥4	≥64	≥64	≥64	8	≥8	≥16	≤1	≥16	64
PA_D22	≤2	≥32	16	8	≥4	≥64	≥64	≥64	8	≥8	≥16	2	≥16	64
*Patient 4*														
PA_D16	4	≥32	≥64	≥64	≥4	≥64	≥64	≥64	≥64	≥8	≥16	≥16	≥16	≥128
PA_D17	4	≥32	≥64	≥64	≥4	≥64	≥64	≥64	≥64	≥8	≥16	≥16	≥16	≥128
PA_D20	≤2	≥32	≥64	16	≥4	≥64	≥64	≥64	32	≥8	≥16	≥16	≥16	32
PA_D23	4	≥32	≥64	16	≥4	≥64	≥64	≥64	16	≥8	≥16	≥16	≥16	16
PA_D24	4	≥32	≥64	16	≥4	≥64	≥64	≥64	16	≥8	≥16	≥16	≥16	16
PA_D25	≤2	≥32	≥64	8	≥4	≥64	≥64	≥64	16	≥8	≥16	≥16	≥16	16

### 2.4. Evolutionary dynamics of *Pseudomonas aeruginosa* genome during short-term ventilator-associated pneumonia infection

We further analysed single-nucleotide polymorphisms (SNPs) and short indels between the earliest isolate and late isolates from each patient by mapping the HiSeq or MiSeq reads of late isolates to the genome of earliest isolate. In addition, we noted that some of the isolates displayed an autolytic colony phenotype, which suggests loss of function of *lasR* [18]. Since our SNP analysis failed to identify *lasR* mutations in some of these isolates, we further investigated if there are any genome rearrangement events that caused the disruption of *lasR*. As expected, genome rearrangement was identified in PA_D9 genome, which causes the disruption of both *lasR* and *mpl* (electronic supplementary material, figure S2). We then designed primers flanking both genes to screen for gene deletions for all the 25 isolates. The PCR results showed that PA_D8, which was isolated one day before PA_D9 from patient 1, probably underwent the same genome rearrangement that disrupted both *lasR* and *mpl* (electronic supplementary material, figure S2). In addition, we noted that PA_D4 and PA_D19 isolated from patient 2 showed deletion of *lasR* but not *mpl* (electronic supplementary material, figure S2). Taken together, we summarized all identified SNPs, short indels and gene deletion events in the 25 isolates in table 2.

**Table 2.** Mutations identified in 25 isolates. SNPs, short indels and gene deletion events are summarized in this table. In each patient, the earliest isolate was chosen as the ancestor for the identification of mutations of the later isolates. For patient 2, the earliest isolate PA_D2 already harbours a non-synonymous mutation in *lasR*. Therefore, the detection of mutations in *lasR* for all the isolates from patient 2 were analysed with PA_D1 as the reference. Bold type indicates convergent evolution events in the *P. aeruginosa* genomes across the four VAP patients.

isolate	time	coding region change	amino acid change	type	non-synonymous	remarks on *lasR* quorum sensing
*patient 1*						
PA_D1	Day 1	—	—	—	—	functional *lasR* quorum sensing
PA_D3	Day 20	***mpl*, 782T > G**	Val261Gly	SNV	yes	functional *lasR* quorum sensing
PA_D8	Day 38	**deletion of *lasR***	—	—	—	*lasR* mutant
		**deletion of *mpl***	—	—	—	
PA_D9	Day 39	**deletion of *lasR***	—	—	—	*lasR* mutant
		**deletion of *mpl***	—	—	—	
*patient 2*						
PA_D2	Day 1	***lasR*, 167A > G (compared to PA_D1)**	Tyr56Cys	SNV	yes	*lasR* mutant
PA_D4	Day 14	**deletion of *lasR***	—	—	—	*lasR* mutant
		***mpl*, 998G > A**	Arg333His	SNV	yes	
		*pelD*, 201T > C	—	SNV	no	
PA_D6	Day 25	***lasR*, 167A > G (compared to PA_D1)**	Tyr56Cys	SNV	yes	*lasR* mutant
PA_D10	Day 29	*mtlB*, 296G > A	Gly99Asp	SNV	yes	functional *lasR* quorum sensing
		*pelD*, 201T > C	—	SNV	no	
PA_D12	Day 50	***mpl*, 998G > A**	Arg333His	SNV	yes	functional *lasR* quorum sensing
		*ampD, 290G* *>* *A*	Gly97Asp	SNV	yes	
		*ampD, 262C* *>* *T*	Gln88*	SNV	yes	
		*pelD*, 201T > C	—	SNV	no	
PA_D19	Day 77	**deletion of *lasR***	—	—	—	*lasR* mutant
		***mpl*, 998G > A**	**Arg333His**	**SNV**	**yes**	
		***pvdS*, 139T > C**	**Phe47Leu**	**SNV**	**yes**	
		hypothetical protein, 286C > T	His96Tyr	SNV	yes	
		hypothetical protein, 42delC	Pro16fs	deletion	yes	
		BarA sensory histidine kinase, *gacS*, 1637T > C	Leu546Pro	SNV	yes	
		multimodular transpeptidase-transglycosylase, 1558T > C′	Ser520Pro	SNV	yes	
		*pelD*, 201T > C	—	SNV	no	
PA_D21	Day 78	***pvdS*, 139T > C**	**Phe47Leu**	**SNV**	**yes**	functional *lasR* quorum sensing
		hypothetical protein, 175A > C	Thr59Pro	SNV	yes	
		*mtlB*, 296G > A	Gly99Asp	SNV	yes	
		*pelD*, 201T > C	—	SNV	no	
*patient 3*						
PA_D5	Day 1	—	—	—	—	functional *lasR* quorum sensing
PA_D7	Day 8	***mpl*, 104_105insC**	Met38fs	insertion	yes	
		***lasR*, 646C > T**	Arg216Trp	SNV	yes	*lasR* mutant
PA_D11	Day 27	***mpl*, 104_105insC**	Met38fs	insertion	yes	*lasR* mutant
		***lasR*, 646C > T**	Arg216Trp	SNV	yes	
		putative lipoprotein, 203A > G	Asp68Gly	SNV	yes	
PA_D13	Day 36	***mpl*, 104_105insC**	Met38fs	insertion	yes	*lasR* mutant
		***lasR*, 646C > T**	Arg216Trp	SNV	yes	
		hypothetical protein, 489G > A	Trp163*	SNV	yes	
PA_D14	Day 43	***mpl*, 104_105insC**	Met38fs	insertion	yes	*lasR* mutant
		***lasR*, 646C > T**	Arg216Trp	SNV	yes	
		*fliK*, 996G > T	—	SNV	no	
PA_D15	Day 50	***mpl*, 104_105insC**	Met38fs	insertion	yes	*lasR* mutant
		***lasR*, 646C > T**	Arg216Trp	SNV	yes	
PA_D18	Day 57	***mpl*, 104_105insC**	Met38fs	insertion	yes	*lasR* mutant
		***lasR*, 646C > T**	Arg216Trp	SNV	yes	
PA_D22	Day 68	***mpl*, 104_105insC**	**Met38fs**	**insertion**	**yes**	*lasR* mutant
		***lasR*, 646C > T**	**Arg216Trp**	**SNV**	**yes**	
		***pvdS*, 77T > C**	**Val26Ala**	**SNV**	**yes**	
*patient 4*						
PA_D16	Day 1	—	—	—	—	functional *lasR* quorum sensing
PA_D17	Day 2	—	—	—	—	functional *lasR* quorum sensing
PA_D20	Day 8	—	—	—	—	functional *lasR* quorum sensing
PA_D23	Day 21	***lasR*, 414delC**	Glu139fs	deletion	yes	*lasR* mutant
		gltJ, 137G > T	Arg46Leu	SNV	yes	
		gltJ, 140G > A	Arg47Gln	SNV	yes	
		gltJ, 146T > G	Leu49Arg	SNV	yes	
		gltJ, 149T > C	Leu50Pro	SNV	yes	
		gltJ, 151T > G	Ser51Ala	SNV	yes	
		predicted signal transduction protein, 596C > T	Pro199Leu	SNV	yes	
		dipeptide-binding ABC transporter, periplasmic substrate-binding component, 1228_1229insACG	Tyr410_Glu411insAsp	Insertion	yes	
PA_D24	Day 24	***lasR*, 414delC**	**Glu139fs**	**deletion**	**yes**	*lasR* mutant
		***pvdS*, 343delC**	**Leu115fs**	**deletion**	**yes**	
PA_D25	Day 25	***lasR*, 414delC**	**Glu139fs**	**deletion**	**yes**	*lasR* mutant
		**non-ribosomal peptide synthetase modules, pyoverdine, 5765_5766insG**	**Ala1925fs**	**insertion**	**yes**	
		*dppC*, 160_161insC	Tyr56fs	insertion	yes	
		DNA primase, phage associated, 2006_2007insG	Ala672fs	insertion	yes	
		polymyxin resistance protein ArnT, undecaprenyl phosphate-alpha-L-Ara4N transferase; Melittin resistance protein PqaB, 647T > C	Leu216Pro	SNV	yes	
		probable sensor/response regulator hybrid, 649G > A	Gly217Arg	SNV	yes	
		NADH:ubiquinone oxidoreductase 49 kD subunit 7, 308C > T	Pro103Leu	SNV	yes	
		probable conserved membrane protein, 284T > C	Leu95Pro	SNV	yes	
		probable iron–sulfur binding protein YPO1417, 213delC	Gly72fs	deletion	yes	
		Kynurenine formamidase, 527T > C	Val176Ala	SNV	yes	
		AraC family transcriptional regulator, 88C > T	Arg30Cys	SNV	yes	
		*potA*, 584C > T	Ala195Val	SNV	yes	
		Enoyl-CoA hydratase, 466C > T	Pro156Ser	SNV	yes	
		3-deoxy-manno-octulosonate cytidylyltransferase, 8A > G	Gln3Arg	SNV	yes	
		*gacA*, 242T > C	Val81Ala	SNV	yes	
		benABC operon transcriptional activator BenR, 866A > G	Asp289Gly	SNV	yes	
		hypothetical protein, 176A > G	Asp59Gly	SNV	yes	
		Alpha-methylacyl-CoA racemase, 841C > T	Arg281Trp	SNV	yes	
		response regulator containing a CheY-like receiver domain and a GGDEF domain, 180G > A	Met60Ile	SNV	yes	
		lysine-specific permease, 449G > A	Gly150Asp	SNV	no	
		putative translation initiation inhibitor, yjgF family, 184A > G	Ser62Gly	SNV	no	
		*dppc*, 160_161insC	Tyr56fs	insertion	yes	
		*priA*, 241C > T	—	SNV	no	

**Figure 3.**
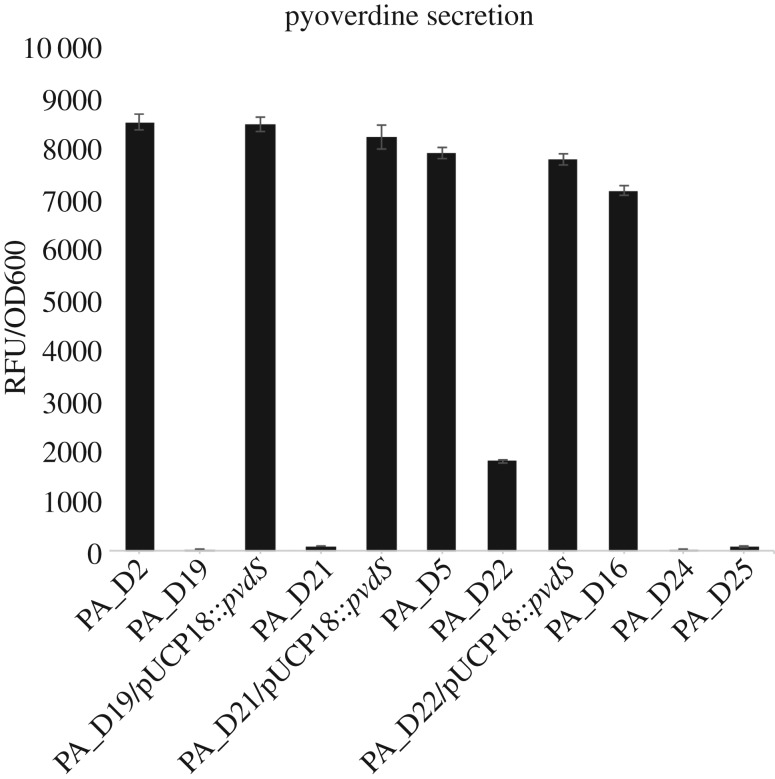
Decreased pyoverdine production by *pvdS* and NPRS mutants. The four *pvdS* mutants (PA_D19, PA_D21, PA_D22 and PA_D24) and PA_D25 produced significantly less pyoverdine compared with their ancestor strains, which do not carry any mutations in genes involved in pyoverdine synthesis pathway. Complementation of PA_D19, PA_D21 and PA_D22 with pUCP18::pvdS restored their pyoverdine production to the similar levels as their ancestors. We could not complement the PA_D24 strain with pUCP18::pvdS due to its high resistance to carbenicillin.

We found that *P. aeruginosa* evolved rapidly in all four VAP patients (table 2). In patient 1, PA_D3 carrying a non-synonymous mutation in *mpl* was isolated 20 days after the onset of VAP infection. At the late stage of infection, PA_D8 and PA_D9, both of which harbour disrupted *lasR* and *mpl* due to genome rearrangements, have replaced PA_D3 and dominated the airway of patient 1 (table 2; electronic supplementary material, figure S2). In patient 2, the earliest isolate PA_D2 already displayed a quorum-sensing negative phenotype, which is probably caused by a non-synonymous mutation in *lasR* (table 2), whereas the latest two isolates PA_D19 and PA_D21 carried a non-synonymous mutation in the *pvdS* gene. In patient 3, non-synonymous mutation in *lasR* and single-nucleotide insertion in *mpl* were first identified in PA_D7 isolated on day 8 and were also carried by all subsequent isolates. The latest isolate PA_D22 from patient 3 acquired an additional non-synonymous mutation in *pvdS* (table 2). In patient 4, the early isolates PA_D16, PA_D17 and PA_D20 were not identified with any mutations in the coding regions of their genomes, whereas a single-nucleotide deletion in *lasR* was identified in PA_D23, PA_D24 and PA_D25 (table 2). It was noted that both PA_D24 and PA_D25 produce much lower levels of pyoverdine (figure 3), which can be explained by a single-nucleotide deletion in *pvdS* of PA_D24, and a single-nucleotide insertion in a gene encoding a non-ribosomal peptide synthetase (NRPS) in the pyoverdine synthesis pathway of PA_D25 [19,20] (table 2).

The ratio of non-synonymous substitution rate (dN) to synonymous substitutions rate (dS) could be used to infer the type of selective force that shapes the population during adaptive evolution; a d*N*/d*S* ratio of greater than 1 suggests positive selection during evolution. In this study, almost all detected mutations are non-synonymous SNPs, and gene deletions (table 2). This provided the direct evidence that strong positive selective forces have dominated the *P. aeruginosa* genomes in the VAP lungs.

### 2.5. Convergent evolution of *Pseudomonas aeruginosa* genome during *in vivo* infection

To investigate whether there are any convergent evolution events among the four patients, we compared the mutations carried by the latest isolates from each patient. The evolution of mutations in *mpl*, which encodes l-alanyl-gamma-d-glutamyl-meso-diaminopimelate ligase [21,22], was identified in the late isolates from patient 1, patient 2 and patient 3. The evolution of mutations in *lasR*, which encodes a major transcriptional activator of the *las* quorum-sensing system [23] was found in the late isolates from all the four patients (table 2). We also identified the evolution of pyoverdine-deficient mutants in patient 2, patient 3 and patient 4 (figure 3). This pyoverdine-deficient phenotype is probably due to mutations of *pvdS,* which encodes a sigma factor that coordinates the expression of multiple proteins involved in pyoverdine synthesis and many virulence factors [19], or a single-nucleotide insertion in a gene predicted to encode an NRPS module required for pyoverdine synthesis pathway [20] (table 2). Specifically, the pyoverdine-deficient mutants were isolated at much later stages of VAP infections than *lasR* and *mpl* mutants (table 2).

To confirm whether the pyoverdine-deficient phenotype of the *pvdS* mutants is due to non-synonymous SNPs in the *pvdS* gene, we cloned and inserted the wild-type *pvdS* gene of the ancestor PA_D1 into pUCP18 vector to construct a pUCP18::*pvdS* plasmid. Complementation of the PA_D19, PA_D21 and PA_D22 with the pUCP18::*pvdS* completely restored their pyoverdine production to the same level as their ancestors, confirming that the non-synonymous SNPs in *pvdS* are responsible for the pyoverdine-deficient phenotype of these mutants (figure 3).

### 2.6. Adaptive evolution of *Pseudomonas aeruginosa* in the lungs of ventilator-associated pneumonia patient leads to attenuated virulence *in vitro* and *in vivo*

**Figure 4.**
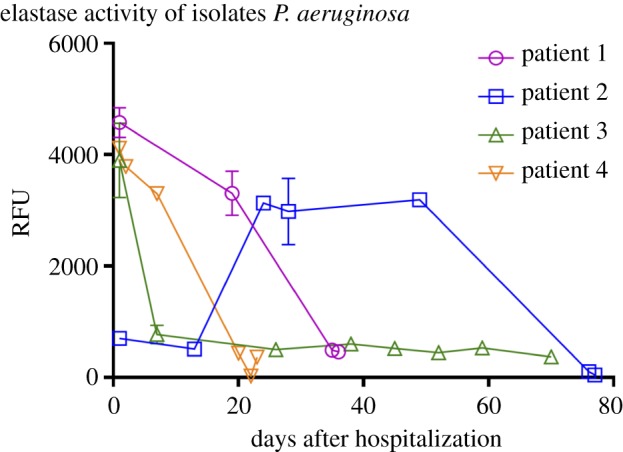
Elastase production of the 25 isolates. Isolates from the same patient were lined and plotted against the time of isolation. In patients 1, 3 and 4, the earliest isolates produced high levels of elastase, whereas the latest isolates produced much lower levels. In patient 2, the earliest two isolates low levels of elastase, whereas the three later isolates produced higher levels and eventually the latest two isolates produced the lowest levels of elastase.

Previous studies showed that both *las* quorum-sensing system and PvdS are required for the expression of various virulence factors, including elastase, rhamnolipids and alkaline proteases [23–27]. The convergent evolution of *P. aeruginosa lasR* and *pvdS* mutants during VAP infections has led us to hypothesize that the late isolates are less virulent compared with their earlier isolated counterparts. To verify this, we first assessed the *in vitro* production of elastase, which is a major virulence factor produced by *P. aeruginosa* [23], for all the 25 isolates. Interestingly, the earliest isolates from patient 1, patient 3 and patient 4 produced the highest levels of elastase, whereas the late isolates have reduced elastase production (figure 4). In patient 2, however, the earliest two isolates produced low levels of elastase, probably due to mutations of *lasR*, whereas the three later isolates produced much higher levels of elastase than the earliest isolate, and eventually the latest two isolates produced the lowest levels (figure 4). These results showed that the late isolates from VAP patient are probably less virulent than their early isolated counterparts.

**Figure 5.**
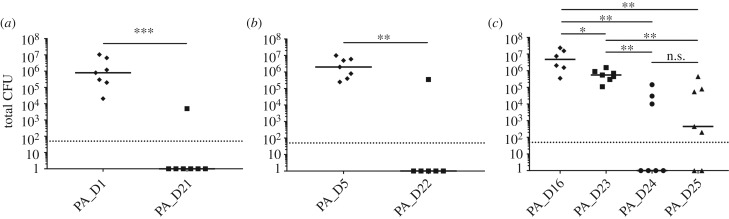
The abilities of early and late isolates to cause acute lung infections in a murine pulmonary infection model. Mice were infected with each strain by intranasal inoculation at 10^6^ CFU per mouse. The total bacterial count recovered from the lungs after 24 h of inoculation are shown in the figure. Comparison between CFU recovered from the lungs of mice infected with (*a*) PA_D1 (ancestor stain from patient 1) and PA_D21 (*pvdS* mutant from patient 2), (*b*) PA_D5 (ancestor stain from patient 3) and PA_D22 (*lasR/pvdS* mutant from patient 3), (*c*) PA_D16 (ancestor stain from patient 4) and PA_D23 (*lasR* mutant from patient 4), PA_D24 (*lasR/pvdS* mutant from patient 4), PA_D25 (*lasR*/NRPS mutant from patient 4). Solid lines: the median for each group. Dotted lines: the detection limit of CFU counting (*n* = 6 for PA_D21 and PA_D16, and *n* = 7 for the other isolates; *p < 0.05, **p < 0.01, ***p < 0.001, n.s. *p* > 0.05; Mann–Whitney test).

Both quorum sensing and pyoverdine were previously shown to be important for *P. aeruginosa* to establish infections *in vivo* [11,28,29]. Therefore, we further employed a murine pulmonary infection model to compare the *in vivo* virulence of PA_D1 with PA_D21 (*pvdS* mutant), PA_D5 with PA_D22 (*lasR*/*pvdS* mutant), and PA_D16 with PA_D23 (*lasR* mutant), PA_D24 (*lasR*/*pvdS* mutant) and PA_D25 (*lasR*/NRPS mutant), in order to investigate if the late isolates are less virulent *in vivo*, and if so, which mutation(s) is the contributing factor. Of note, we cross-compared PA_D1 and PA_D21 because the ancestor strain PA_D2 from patient 2 already harbours a *lasR* mutation. These strains were administrated intranasally at 10^6^ colony forming units (CFU) per mouse. After 24 h, the total number of bacteria residing in the lungs was numerated to assess the severity of pulmonary infection. As expected, the three ancestor strains PA_D1, PA_D5 and PA_D16 caused severe pulmonary infections in mice (figure 5). Intriguingly, the *lasR* mutant PA_D23 only showed a mild decrease in CFU compared with its ancestor PA_D16, whereas the *pvdS* mutant PA_D21 showed a drastic decrease in CFU compared with the wild-type PA_D1 (figure 5). Furthermore, the *lasR/pvdS* double mutants PA_D22 and PA_D24, and the *lasR*/NRPS double mutant PA_D25, all displayed the similar phenotype as the *pvdS* single mutant PA_D21 (figure 5). Taken together, these results confirmed that the late isolates are less virulent *in vivo* compared with the early isolates, which can largely be explained by mutations in *pvdS* or NRPS, and/or to a much smaller but significant extent, by mutations in *lasR*.

## 3. Discussion

In this study, we used both PacBio and Illumina sequencing to obtain the complete and draft genomes of 25 *P. aeruginosa* clinical isolates belonging to one monophyletic group. Clinical isolates from this group showed multidrug resistance and are responsible for VAP infections in a teaching hospital in China. We showed that these isolates may have acquired various antibiotic resistance genes, many of which are probably acquired through horizontal gene transfer events. This is evidenced by the fact that most of the acquired antibiotic resistance genes are in the GIs (figure 2). Furthermore, we also studied the *in vivo* evolution of this strain during VAP infections using the high-quality Illumina reads. The time scale for our *in vivo* evolutionary study is within a period of 78 days, whereas previous studies investigating the genetic adaptation and evolution of *P. aeruginosa* in the host mainly focused on lung infection in CF patients and the time scale for isolates sampling ranged from six months to several decades [10,30–34]. Our results showed that positive selection has shaped the *P. aeruginosa* genome and led to three convergent evolution events, which are the evolution of mutations in *mpl* and *lasR* and a pyoverdine-deficient phenotype.

In *P. aeruginosa*, the *mpl* mutants were shown to overexpress *ampC*, which leads to a higher resistance to β-lactam antibiotics such as penicillin and cephalosporins [21,22]. The deletion of *mpl* from *P. aeruginosa* was reported to cause an approximately 20-fold increase in the β-lactamase activity, which led to a 1.5-fold increase in ceftazidime MIC [21]. The association between mutation of *mpl* and the increased β-lactam antibiotic resistance was also observed in our results (tables 1 and 2). For example, in patient 1, the two *mpl* deletion mutants PA_D8 and PA_D9, as well as PA_D3 carrying a non-synonymous mutation in *mpl* showed a fourfold increase in ceftazidime MIC compared with their ancestor PA_D1. In patient 3, except for the ancestor strain PA_D5, all the other isolates carried a single-nucleotide insertion in *mpl*. These mutants all showed at least twofold increase of ceftazidime MIC and fourfold increase of piperacillin-tazobactam MIC compared with PA_D5. In patient 2, both PA_D4 and PA_D19 carry the same non-synonymous mutation in *mpl*, resulting in an amino acid change Arg333His. In these two mutants, we did not observe any increase in β-lactam MICs, probably because this amino acid change does not significantly affect the function of Mpl. Interestingly, the day 50 isolate PA_D12 that harbours a single-nucleotide insertion in the *ampD* gene showed a fourfold increase of ceftazidime MIC and a more than twofold increase of piperacillin-tazobactam MIC compared with PA_D2. Similar to *mpl* mutants, the *ampD* mutant was also reported to overexpress *ampC*, which leads to increased resistance to β-lactam antibiotics [21,35]. It is possible that the increased resistance of PA_D12 to both ceftazidime and piperacillin-tazobactam is resulted from the inactivation of the *ampD* gene. Taken together, the evolution of *mpl* and *ampD* mutants in VAP patients is probably due to the selective pressure imposed by intensive β-lactam treatments.

Like *mpl*, gene deletions, single-nucleotide insertions and two types of non-synonymous mutations were identified in the *lasR* gene of 16 isolates (table 2). PA_D8 and PA_D9 from patient 1, and PA_D4 and PA_D19 from patient 2, have *lasR* gene deleted from their genomes, whereas all the *lasR* mutants from patient 4 carry a single-nucleotide deletion in *lasR*. These seven isolates probably have completely abolished LasR function. The two *lasR* mutants from patient 2, PA_D2 and PA_D6 synthesize a Try56Cys LasR, which probably has a defective function because the highly conserved Try56 is responsible for LasR to form hydrogen bond with its autoinducer [36]. The seven *lasR* mutants isolated from patient 3 harbour a Arg216Trp mutation in LasR. Of note, the same type of LasR mutation was reported to emerge during the *in vitro* growth of *P. aeruginosa* in rich medium [18]. Arg216 is a conserved residue that lies in α-helix 9 of LasR, which is essential for DNA recognition by LuxR-type transcriptional factors [37]. Mutations at residues Try56 and Arg216 probably affect the ligand binding and target DNA recognition of the LasR protein, respectively. Therefore, we conclude that all the identified *lasR* mutants in this study have severely impaired, if not completely abolished LasR function.

The five pyoverdine-deficient mutants were isolated from three patients at very late stages of VAP infections (table 2). These mutants harbour either non-synonymous mutations (PA_D19, PA_D21 and PA_D22), a single-nucleotide deletion in *pvdS* (PA_D24), or a single-nucleotide insertion in a 14 976 bp gene encoding an NRPS module required for pyoverdine synthesis (PA_D25) (table 2). In *P. aeruginosa*, loss of function of PvdS or NRPS was reported to abolish its pyoverdine biosynthesis, which usually results in a pyoverdine-deficient phenotype [20,38]. As expected, indel mutations in *pvdS* and the NRPS gene probably have completely abolished the functions of the two genes, resulting in complete loss of pyoverdine production in both PA_D24 and PA_D25. The non-synonymous *pvdS* 139T > C mutation harboured by PA_D19 and PA_D21 causes a mutation at Phe47, which is a highly conserved residue in the region 2.1 of sigma factors; this mutation was shown to drastically affect the function of PvdS [39]. By contrast, the PvdS Val26Ala mutation carried by PA_D22 does not happen at an essential amino acid residue, but rather adjacent to a conserved residue Leu25 [39]. Therefore, although PA_D22 showed a reduced level of pyoverdine production than its ancestor PA_D5, it still produces a higher amount of pyoverdine than the other three *pvdS* mutants (figure 3). Nevertheless, complementation of all the three *pvdS* mutants carrying non-synonymous SNPs with wild-type *pvdS* have fully restored their pyoverdine production to the same level as their ancestors, confirming that pyoverdine-deficient phenotype is due to mutations in *pvdS* (figure 3). Previous studies have reported the isolation of pyoverdine-deficient mutants from CF lungs, and the chance of isolating pyoverdine-deficient mutants seemed to increase upon the increment of patient age [9,40,41]. We propose that the convergent evolution of pyoverdine-deficient mutants is possibly due to three reasons. First, the pyoverdine-deficient strains could acquire iron from alternative sources to compensate for the defective ferric iron uptake in the patients’ airways. Second, the reduced pyoverdine synthesis of these mutants may lead to reduced virulence, which may result in less immune attack on pyoverdine-deficient mutants from the host, and hence, enriched pyoverdine-deficient mutants at the infection sites. Third, reduced pyoverdine synthesis has significantly reduced metabolic cost in these mutants, which may associate with better fitness in the host environment.

The pyoverdine-deficient mutants were reported to be deficient in producing exotoxin A and PrpL proteases [42], which are important virulence factors produced by *P. aeruginosa*. Similarly, loss-of-function mutations of *lasR* also reduce the production of many virulence factors such as exotoxin A, elastase, rhamnolipids and pyocyanin in *P. aeruginosa* [23–27]. The convergent evolution of *lasR* and pyoverdine-deficient mutants in VAP patients suggested that the short-term *in vivo* evolution selected less virulent *P. aeruginosa* mutants. In agreement with this, the late isolates did produce lower levels of a major virulence factor elastase *in vitro* compared with the earlier isolated counterparts (figure 4). We also compared the *in vivo* virulence of *lasR* and pyoverdine-deficient mutants using a murine pulmonary infection model. Surprisingly, we found that mutations causing the pyoverdine-deficient phenotype greatly impaired the *in vivo* virulence of *P. aeruginosa*, whereas *lasR* mutation has a much milder effect (figure 5). These findings are in line with previous studies showing that both intact *lasR* function and pyoverdine synthesis are important for *in vivo* virulence [11,28,29]. In addition, our results also provide a direct comparison on the *in vivo* virulence of the two types of mutants isolated from clinical sources.

In conclusion, we identified a novel *P. aeruginosa* strain responsible for a nosocomial outbreak of VAP infections in China. We showed that this strain could undergo rapid adaptive evolution during VAP infections, which led to increased resistance to β-lactams, a defective quorum-sensing system, reduced production of pyoverdine, and attenuated virulence *in vitro* and *in vivo*. Our findings provide novel insights into the short-term evolution of *P. aeruginosa* in the human airways during acute VAP infections.

## 4. Methods

### 4.1. Isolate collection and characterization

All *P. aeruginosa* isolates were collected from clinical specimens. Sputum samples from VAP patients were streaked onto blood agar plate and incubated at 35°C for 18 h. Three colonies from each cultivation were randomly selected and sub-cultured in LB broth and stored in 25% glycerol under −80°C. The 16S rRNA gene of each isolate was amplified using primers 27F (5′-GAGTTTGATCCTGGCTCAG-3′) and 1492R (5′-GGTTACCTTGTTACGACTT-3′) [43]. PCR products were purified and sequenced to identify the species of each isolate. RAPD typing was performed using primer 272 as previously described [44]. Susceptibilities to various antibiotics were determined by the VITEK 2 Compact system (bioMérieux).

### 4.2. Sequencing, assembly and annotation

Genomic DNA from a single colony of each isolate was purified using Blood and Cell Culture DNA Midi Kit (Qiagen) and sequenced on PacBio RS II platform, Illumina HiSeq 2500 platform or Illumina MiSeq platform. The sequencing data were used to assemble the full-length genomes and identify SNPs in this study. All sequencing data used in this study are available on NCBI registered under BioProject no. PRJNA294254. The full-length genome of eight isolates was assembled from long reads obtained from PacBio RS II system using HGAP3 pipeline. The assembled genomes were then corrected with high-quality Illumina HiSeq reads using CLC Genomic Workbench 8.5 (CLC Bio, Qiagen) assisted with manual curation to further minimize sequencing errors and close gaps in the assembled genomes. The corrected full-genome files were uploaded to the Rapid Annotations using Subsystem Technology (RAST) server for bacterial genome annotation [14].

### 4.3. Comparative genomics and phylogenetic analysis

Core-genome alignment was carried out using Parsnp v. 1.1.2 [45], yielding an alignment with 4 561 475 putatively homologous sites. Of these, 136 126 sites were found to contain potential single-nucleotide variants. Recombinant sites in the alignment were then identified with PhiPack [46] (version integrated with the Parsnp). In total, 132 511 variant sites remained after removing putative recombinant and low-quality variant sites. Phylogenetic inference was then carried out on the variant sites using the approximate maximum-likelihood algorithm implemented in FastTree2 (version integrated with Parsnp), with clade confidence estimated with SH-like support values [47]. Finally, branch lengths in the inferred tree were rescaled from substitutions per variant site to represent substitutions per core-genome site. Genomic islands of PA_D1 genome were predicted by IslandViewer 3 server [15]. Antibiotic resistance genes were predicted from the 25 assembled genomes were done using the ResFinder 2.1 server [16]. Comparison of PA_D1 genome against other five full *P. aeruginosa* genomes was done by BLAST search using BLAST Ring Image Generator 0.95 [48], with locations of genomic islands and antibiotic resistance genes marked in the output image as instructed by the manual.

### 4.4. Detection of nucleotide differences

Nucleotide differences were detected and evaluated using CLC Genomic Workbench 8.5. Paired-end reads in FASTQ formats were mapped to the annotated PA_D1 reference genome and further compared with each other to generate lists of SNPs and short indels. A frequency cut-off of more than 80% was set to minimize false SNPs due to sequencing error. PCR was used to verify detected gene deletions. Genome rearrangements were detected by ProgressiveMauve v. 2.3.1 [49]. Primers lasR_F (5′-GGAATTCCTTCTCGGACTGCCGTACAAC-3′) and lasR_R (5′-GCTCTAGAGCAAATTACCGATCGCCAG-3′) were used to amplify the entire coding region of *lasR*, whereas mpl_F (5′-CAACACCCTGTATCGCAAGC-3′) and mpl_R (5′-ATCGCCAGGGTAATGCGTTC-3′) were used to amplify *mpl*. PCR products were resolved in 1% agarose gel. The sizes of the amplified products are 865 bp and 1608 bp, respectively.

### 4.5. Quantification of elastase and pyoverdine

Bacterial strains were grown in ABT minimal medium [50] supplemented with 5 g l^–1^ glucose and 2 g l^–1^ casamino acids. The filtered supernatants of overnight culture were used to quantify the productions of elastase and pyoverdine *in vitro*. The quantification of elastase production was performed in a 96-well plate using EnzCheck Elastase Assay Kit (Thermo Fisher Scientific) as instructed by the manual. Elastase activity was measured by Infinite M200 PRO system (Tecan) and the results were shown as emission at 530 nm. For the quantification of pyoverdine, filtered supernatant was measured for fluorescence signal emitted at 460 nm upon excitation at 398 nm using Infinite M200 PRO system (Tecan). Both experiments were performed in at least triplicates and the results are shown as mean ± standard deviation in the figures.

### 4.6. *Pvds* complementation

The wild-type pvdS gene was amplified from PA_D1 genomic DNA using primers pvdS_EcoRI_F (5′-CCGAATTCCGCAGCAAGGTGATTTCCAT-3′) and pvdS_KpnI_R (5′-CCGGTACCCTGCGAGAAGGAGTTCGACT-3′), and inserted into EcoRI and KpnI sites in pUCP18 vector to construct *pvdS* complementation plasmid pUCP18::*pvdS*. The pUCP18::*pvdS* plasmid was transformed into PA_D19, PA_D21 and PA_D22 by electroporation.

### 4.7. Animal model

A murine pulmonary infection model was used to evaluate the virulence of *P. aeruginosa* strains. Briefly, bacterial cells of overnight culture were washed three times and re-suspended in phosphate-buffered saline. Bacterial suspensions were administrated intranasally to each mouse at 20 µl per mouse under anaesthesia (approx. 1 × 10^6^ CFU per mouse). After 24 h, all mice were sacrificed and the lungs were harvested and kept in ice-cold 0.9% NaCl saline. The bacterial cells residing in the lungs were suspended into the 0.9% NaCl saline by homogenization using a Bio-Gen PRO200 Homogenizer (Pro Scientific). CFU was quantified by serial dilution and plating on LB agar.

### 4.8. Nucleotide sequence accession numbers

The whole-genome Illumina shotgun sequencing for *P. aeruginosa* PA_D1 to PA_D25 has been deposited at GenBank under the accession numbers SRX2066370, SRX2066389, SRX2066394, SRX2066397, SRX2066399, SRX2066406, SRX2066408, SRX2066409, SRX2066415, SRX2066423, SRX2066444, SRX2066449, SRX2066453, SRX2066456, SRX2066475, SRX2066480, SRX2066484, SRX2066486, SRX2066488, SRX2066489, SRX2066521, SRX2066560, SRX2066602, SRX2066605 and SRX2066607. The whole-genome shotgun sequencing on Pacbio RS II platform for the 8 isolates are deposited under accession numbers SRX2066384, SRX2066390, SRX2066402, SRX2066416, SRX2066482, SRX2066542, SRX2066581 and SRX2066616.

## Supplementary Material

Supplementary Figures and Tables
