# Supplementary material for: The rapid in vivo evolution of Pseudomonas aeruginosa in ventilator-associated pneumonia patients leads to attenuated virulence
Source: Open Biol. 2017 Sep 6;7(9):170029. doi: 10.1098/rsob.170029 (PMC5627047; doi:10.1098/rsob.170029)

**The rapid *in vivo* evolution of *Pseudomonas aeruginosa* in ventilator-associated pneumonia patients leads to attenuated virulence**

Ke Wang ^1,2^, Yi-qiang Chen ^1*^, May M. Salido ^3^, Gurjeet S. Kohli ^3^, Jin-liang Kong ^1^, Hong-jie Liang ^4^, Zi-ting Yao ^2^, Yan-tong Xie ^5^, Hua-yu Wu ^6^, Shuang-qi Cai ^1^, Daniela I. Drautz-Moses ^3^, Aaron E. Darling ^7^, Stephan C. Schuster ^3,8^, Liang Yang ^3,8^, Yichen Ding ^3,8,9*^

^1^Department of Respiratory Disease, First Affiliated Hospital of Guangxi Medical University, Nanning, Guangxi, China, 530021

^2^Centre for Genomic and Personalized Medicine, Guangxi Medical University, Nanning, Guangxi, China, 530021

^3^Singapore Centre for Environmental Life Sciences Engineering (SCELSE), Nanyang Technological University, Singapore 637551

^4^Department of Clinical Laboratory, First Affiliated Hospital of Guangxi Medical University, Nanning, Guangxi, China, 530021

^5^The First Clinical School of Guangxi Medical University, Nanning, Guangxi, China, 530021

^6^Department of Cell Biology and Genetics, Guangxi Medical University, Nanning, Guangxi, China, 530021

^7^The ithree Institute, University of Technology Sydney, Sydney, NSW, Australia

^8^School of Biological Sciences, Nanyang Technological University, Singapore 637551

^9^Interdisciplinary Graduate School, SCELSE, Nanyang Technological University, Singapore 639798

*Corresponding authors:

Mr. Yichen Ding

Singapore Centre for Environmental Life Sciences Engineering,

Nanyang Technological University,

Singapore 637551

Tel: +65 98900605

Fax: +65 63167349

Email: [DI0001EN@e.ntu.edu.sg](mailto:DI0001EN@e.ntu.edu.sg)

Dr. Yi-qiang Chen

Department of Respiratory Disease,

First Affiliated Hospital of Guangxi Medical University,

Nanning, 530000, P. R. China

Tel: +86-771-5350993

Fax: +86-771-5350993

Email:chenyq0708@foxmail.com

**Running title:** *In vivo* evolution of *P. aeruginosa*

**Table S1** **Isolate IDs, isolation date and patient information.** Isolates were grouped in the table according to their isolation sources. Patient information and the diagnosed conditions were also listed in the table.

| **Isolate ID** | **Isolation date** | **Source** | **Diagnosed conditions of the patient** |
| --- | --- | --- | --- |
| **Patient 1 (Male, 50y)** | | | |
| PA_D1 | 14-Dec-13 | sputum | Diabetes, brain herniation, ventilator-associated pneumonia |
| PA_D3 | 2-Jan-14 | sputum |  |
| PA_D8 | 20-Jan-14 | sputum |  |
| PA_D9 | 21-Jan-14 | sputum |  |
| **Patient 2 (Male, 76y)** | | | |
| PA_D2 | 24-Dec-13 | sputum | Cerebral hemorrhage, ventilator-associated pneumonia |
| PA_D4 | 6-Jan-14 | sputum |  |
| PA_D6 | 17-Jan-14 | sputum |  |
| PA_D10 | 21-Jan-14 | sputum |  |
| PA_D12 | 11-Feb-14 | sputum |  |
| PA_D19 | 10-Mar-14 | sputum |  |
| PA_D21 | 11-Mar-14 | sputum |  |
| **Patient 3 (Male, 82y)** | | | |
| PA_D5 | 13-Jan-14 | sputum | Diabetes, peripheral lung cancer, ventilator-associated pneumonia |
| PA_D7 | 20-Jan-14 | sputum |  |
| PA_D11 | 8-Feb-14 | sputum |  |
| PA_D13 | 17-Feb-14 | sputum |  |
| PA_D14 | 24-Feb-14 | sputum |  |
| PA_D15 | 3-Mar-14 | sputum |  |
| PA_D18 | 10-Mar-14 | sputum |  |
| PA_D22 | 21-Mar-14 | sputum |  |
| **Patient 4 (Male, 46y)** | | | |
| PA_D16 | 6-Mar-14 | sputum | Nasopharyngeal cancer, ventilator-associated pneumonia |
| PA_D17 | 7-Mar-14 | sputum |  |
| PA_D20 | 13-Mar-14 | sputum |  |
| PA_D23 | 26-Mar-14 | sputum |  |
| PA_D24 | 29-Mar-14 | sputum |  |
| PA_D25 | 30-Mar-14 | sputum |  |

**Table S2 General characteristics of the 8 fully sequenced *P. aeruginosa* genomes.**

|  | **PA_D1** | **PA_D2** | **PA_D5** | **PA_D9** | **PA_D16** | **PA_D21** | **PA_D22** | **PA_D25** |
| --- | --- | --- | --- | --- | --- | --- | --- | --- |
| **Length (bp)** | 6,643,823 | 6,642,996 | 6,681,992 | 6,645,477 | 6,681,975 | 6,639,108 | 6,681,981 | 6,683,204 |
| **G+C content** | 66.2% | 66.2% | 66.2% | 66.2% | 66.2% | 66.2% | 66.2% | 66.2% |
| **Genes** | 6,210 | 6,211 | 6,233 | 6,215 | 6,275 | 6,211 | 6,241 | 6,250 |
| **Protein-coding genes** | 6,135 | 6,136 | 6,158 | 6,140 | 6,160 | 6,136 | 6,166 | 6,175 |
| **tRNA** | 63 | 63 | 63 | 63 | 63 | 63 | 63 | 63 |
| **rRNA** | 12 | 12 | 12 | 12 | 12 | 12 | 12 | 12 |

**Table S3 Genomes used in the phylogenetic analysis.** The genomes used to create phylogenetic tree in Figure 1 are listed in this table, together with their Genbank accession numbers.

| **Genome** | **Accession no.** |
| --- | --- |
| *P. aeruginosa CU1510* | CP013144.1 |
| *P. aeruginosa B136-33* | NC_020912.1 |
| *P. aeruginosa VRFPA04* | NZ_CP008739.1 |
| *P. aeruginosa NCGM257* | NZ_AP014651.1 |
| *P. aeruginosa UCBPP-PA14* | NC_008463.1 |
| *P. aeruginosa M1608* | NZ_CP008862.1 |
| *P. aeruginosa MTB-1* | NC_023019.1 |
| *P. aeruginosa H47921* | NZ_CP008861.1 |
| *P. aeruginosa PA1* | NC_022808.2 |
| *P. aeruginosa PAO1* | NC_002516.2 |
| *P. aeruginosa 122-4-4-(59)* | NZ_CP013696.1 |
| *P. aeruginosa ATCC27853* | NZ_CP015117.1 |
| *P. aeruginosa F22031* | NZ_CP007399.1 |
| *P. aeruginosa VA-134* | NZ_CP013245.1 |
| *P. aeruginosa YL84* | NZ_CP007147.1 |
| *P. aeruginosa NCTC10332* | NZ_LN831024.1 |
| *P. aeruginosa M18* | NC_017548.1 |
| *P. aeruginosa DK2* | NC_018080.1 |
| *P. aeruginosa SJTD-1* | NZ_CP015877.1 |
| *P. aeruginosa RP73* | NC_021577.1 |
| *P. aeruginosa SCV20265* | NC_023149.1 |
| *P. aeruginosa F9676* | NZ_CP012066.1 |
| *P. aeruginosa AES-1R* | NZ_CP013680.1 |
| *P. aeruginosa LES431* | NC_023066.1 |
| *P. aeruginosa DHS01* | NZ_CP013993.1 |
| *P. aeruginosa T63266* | NZ_CP008868.1 |
| *P. aeruginosa N17-1* | NZ_CP014948.1 |
| *P. aeruginosa F30658* | NZ_CP008857.1 |
| *P. aeruginosa Carb01-63* | NZ_CP011317.1 |
| *P. aeruginosa FRD-1* | NZ_CP010555.1 |

**Table S4 GIs in PA_D1 identified by IslandViewer 3.**

| **Island start** | **Island end** | **Length** | **Gene start** | **Gene end** | **Strand** | **Product** |
| --- | --- | --- | --- | --- | --- | --- |
| 40189 | 58316 | 18127 | 40189 | 40404 | 1 | Dodecin (COG3360) Flavin-binding |
| 40189 | 58316 | 18127 | 40588 | 40815 | 1 | hypothetical protein |
| 40189 | 58316 | 18127 | 41112 | 42800 | 1 | Channel-forming transporter/cytolysins activator of TpsB family |
| 40189 | 58316 | 18127 | 42913 | 53478 | 1 | Putative large exoprotein involved in heme utilization or adhesion of ShlA/HecA/FhaA family |
| 40189 | 58316 | 18127 | 55653 | 55922 | 1 | Transposase and inactivated derivatives |
| 40189 | 58316 | 18127 | 57651 | 58046 | 1 | FIG00953153: hypothetical protein |
| 40189 | 58316 | 18127 | 58083 | 58319 | -1 | FIG00954700: hypothetical protein |
| 1210284 | 1214685 | 4401 | 1210284 | 1210595 | 1 | hypothetical protein |
| 1210284 | 1214685 | 4401 | 1212622 | 1214685 | -1 | C-5 cytosine-specific DNA methylase |
| 1210284 | 1214685 | 4401 | 1214682 | 1215227 | -1 | COG1896: Predicted hydrolases of HD superfamily |
| 1226538 | 1240912 | 14374 | 1227676 | 1228332 | 1 | hypothetical protein |
| 1226538 | 1240912 | 14374 | 1228586 | 1229161 | 1 | FIG00956602: hypothetical protein |
| 1226538 | 1240912 | 14374 | 1229164 | 1231173 | 1 | Phage terminase, large subunit |
| 1226538 | 1240912 | 14374 | 1231185 | 1231409 | 1 | hypothetical protein |
| 1226538 | 1240912 | 14374 | 1231409 | 1232875 | 1 | Phage portal protein, lambda family |
| 1226538 | 1240912 | 14374 | 1232859 | 1234031 | 1 | Prophage Clp protease-like protein |
| 1226538 | 1240912 | 14374 | 1234028 | 1234663 | 1 | hypothetical protein |
| 1226538 | 1240912 | 14374 | 1234731 | 1235729 | 1 | elements of external origin; phage-related functions and prophages |
| 1226538 | 1240912 | 14374 | 1235732 | 1236043 | 1 | hypothetical protein |
| 1226538 | 1240912 | 14374 | 1236040 | 1236477 | 1 | hypothetical protein |
| 1226538 | 1240912 | 14374 | 1236528 | 1236728 | 1 | hypothetical protein |
| 1226538 | 1240912 | 14374 | 1236756 | 1237502 | 1 | hypothetical protein |
| 1226538 | 1240912 | 14374 | 1237735 | 1238175 | 1 | hypothetical protein |
| 1226538 | 1240912 | 14374 | 1238323 | 1238748 | -1 | hypothetical protein |
| 1226538 | 1240912 | 14374 | 1238782 | 1241664 | 1 | Mu-like prophage FluMu protein gp42 |
| 1240919 | 1252390 | 11471 | 1238782 | 1241664 | 1 | Mu-like prophage FluMu protein gp42 |
| 1240919 | 1252390 | 11471 | 1241661 | 1242059 | 1 | Phage protein |
| 1240919 | 1252390 | 11471 | 1242108 | 1245659 | 1 | PUTATIVE TRANSMEMBRANE PROTEIN |
| 1240919 | 1252390 | 11471 | 1246430 | 1246666 | 1 | hypothetical protein |
| 1240919 | 1252390 | 11471 | 1246675 | 1247523 | 1 | hypothetical protein |
| 1240919 | 1252390 | 11471 | 1247775 | 1249280 | 1 | hypothetical protein |
| 1240919 | 1252390 | 11471 | 1249280 | 1249516 | 1 | hypothetical protein |
| 1240919 | 1252390 | 11471 | 1249762 | 1250391 | 1 | Lytic enzyme |
| 1240919 | 1252390 | 11471 | 1250388 | 1250756 | 1 | FIG00959580: hypothetical protein |
| 1240919 | 1252390 | 11471 | 1250753 | 1251013 | 1 | hypothetical protein |
| 1240919 | 1252390 | 11471 | 1251158 | 1251961 | 1 | Methyl-directed repair DNA adenine methylase (EC 2.1.1.72) |
| 1240919 | 1252390 | 11471 | 1252278 | 1252961 | -1 | Gifsy-2 prophage protein |
| 1484952 | 1489505 | 4553 | 1484952 | 1486211 | -1 | Integrase |
| 1484952 | 1489505 | 4553 | 1486502 | 1486948 | -1 | FIG00962931: hypothetical protein |
| 1484952 | 1489505 | 4553 | 1486945 | 1488780 | -1 | DNA cytosine methyltransferase family protein |
| 1484952 | 1489505 | 4553 | 1489266 | 1489505 | -1 | hypothetical protein |
| 1484952 | 1489505 | 4553 | 1489502 | 1490128 | -1 | hypothetical protein |
| 1648225 | 1656910 | 8685 | 1648752 | 1649588 | 1 | Hydroxymethylpyrimidine ABC transporter, ATPase component |
| 1648225 | 1656910 | 8685 | 1649629 | 1650636 | 1 | ABC-type nitrate/sulfonate/bicarbonate transport systems, periplasmic components |
| 1648225 | 1656910 | 8685 | 1650633 | 1651409 | 1 | ABC-type nitrate/sulfonate/bicarbonate transport system, permease component |
| 1648225 | 1656910 | 8685 | 1651415 | 1652176 | 1 | 3-oxoacyl-[acyl-carrier protein] reductase (EC 1.1.1.100) |
| 1648225 | 1656910 | 8685 | 1652190 | 1652720 | 1 | Mannose-6-phosphate isomerase (EC 5.3.1.8) |
| 1648225 | 1656910 | 8685 | 1652729 | 1653598 | 1 | Alpha/beta hydrolase |
| 1648225 | 1656910 | 8685 | 1653591 | 1654424 | 1 | Transcriptional regulator, IclR family |
| 1648225 | 1656910 | 8685 | 1654411 | 1655208 | 1 | 3-oxoacyl-[acyl-carrier protein] reductase (EC 1.1.1.100) |
| 1648225 | 1656910 | 8685 | 1655201 | 1656883 | 1 | Acetolactate synthase large subunit (EC 2.2.1.6) |
| 1648225 | 1656910 | 8685 | 1656894 | 1657697 | 1 | Aspartate dehydrogenase homolog |
| 2025479 | 2045461 | 19982 | 2025479 | 2026588 | 1 | Biosynthetic Aromatic amino acid aminotransferase beta (EC 2.6.1.57) |
| 2025479 | 2045461 | 19982 | 2026581 | 2028821 | 1 | Cyclohexadienyl dehydrogenase (EC 1.3.1.12)(EC 1.3.1.43) / 5-Enolpyruvylshikimate-3-phosphate syntha |
| 2025479 | 2045461 | 19982 | 2028821 | 2029510 | 1 | Cytidylate kinase (EC 2.7.4.25) |
| 2025479 | 2045461 | 19982 | 2029778 | 2031457 | 1 | SSU ribosomal protein S1p |
| 2025479 | 2045461 | 19982 | 2031594 | 2031878 | 1 | Integration host factor beta subunit |
| 2025479 | 2045461 | 19982 | 2037484 | 2037894 | 1 | UDP-N-acetylglucosamine 4,6-dehydratase (EC 4.2.1.-) |
| 2025479 | 2045461 | 19982 | 2039958 | 2040164 | 1 | UDP-N-acetylglucosamine 2-epimerase (EC 5.1.3.14) |
| 2025479 | 2045461 | 19982 | 2040476 | 2041384 | 1 | Putative glycosyltransferase |
| 2025479 | 2045461 | 19982 | 2043599 | 2045461 | 1 | nucleotide sugar epimerase/dehydratase WbpM |
| 2443375 | 2450503 | 7128 | 2443356 | 2444558 | 1 | Putative phage terminase |
| 2443375 | 2450503 | 7128 | 2444561 | 2445916 | 1 | 62kDa structural protein |
| 2443375 | 2450503 | 7128 | 2445913 | 2446992 | 1 | Phage protein |
| 2443375 | 2450503 | 7128 | 2447089 | 2447859 | 1 | FIG00640540: hypothetical protein |
| 2443375 | 2450503 | 7128 | 2447869 | 2448840 | 1 | probable phage protein YPO2110 |
| 2443375 | 2450503 | 7128 | 2448882 | 2449367 | 1 | hypothetical protein |
| 2443375 | 2450503 | 7128 | 2449351 | 2449815 | 1 | Phage protein |
| 2443375 | 2450503 | 7128 | 2449983 | 2450204 | 1 | hypothetical protein |
| 2443375 | 2450503 | 7128 | 2450208 | 2450882 | 1 | FIG00953886: hypothetical protein |
| 2457633 | 2462168 | 4535 | 2457007 | 2457768 | 1 | Phage tail assembly protein |
| 2457633 | 2462168 | 4535 | 2460638 | 2461042 | -1 | hypothetical protein |
| 2457633 | 2462168 | 4535 | 2461383 | 2461523 | 1 | hypothetical protein |
| 2457633 | 2462168 | 4535 | 2461588 | 2462415 | 1 | Phage antirepressor protein |
| 2532472 | 2538394 | 5922 | 2531539 | 2532507 | -1 | Mycobacteriophage Barnyard protein gp56 |
| 2532472 | 2538394 | 5922 | 2532602 | 2532874 | -1 | FIG00957676: hypothetical protein |
| 2532472 | 2538394 | 5922 | 2534990 | 2537038 | 1 | FIG00483682: hypothetical protein |
| 2532472 | 2538394 | 5922 | 2538363 | 2539748 | -1 | Phage-related integrase |
| 2876509 | 2882483 | 5974 | 2876969 | 2879740 | -1 | FIG00961420: hypothetical protein |
| 2876509 | 2882483 | 5974 | 2879755 | 2880462 | -1 | Rhs family protein |
| 2876509 | 2882483 | 5974 | 2881431 | 2882051 | -1 | Rhs family protein |
| 3541385 | 3545928 | 4543 | 3541788 | 3545048 | 1 | hypothetical protein |
| 4153496 | 4158138 | 4642 | 4151930 | 4154152 | -1 | lipase family protein |
| 4153496 | 4158138 | 4642 | 4154164 | 4154958 | -1 | lipoprotein |
| 4153496 | 4158138 | 4642 | 4155102 | 4155893 | -1 | lipoprotein |
| 4153496 | 4158138 | 4642 | 4155890 | 4156738 | -1 | hypothetical protein |
| 4153496 | 4158138 | 4642 | 4156735 | 4158810 | -1 | VgrG protein |
| 4161975 | 4169100 | 7125 | 4161975 | 4162259 | -1 | FIG00953120: hypothetical protein |
| 4161975 | 4169100 | 7125 | 4162735 | 4163325 | 1 | Adenylylsulfate kinase (EC 2.7.1.25) |
| 4161975 | 4169100 | 7125 | 4164627 | 4164971 | -1 | probable glycosyl transferase |
| 4161975 | 4169100 | 7125 | 4165044 | 4166507 | 1 | hypothetical protein |
| 4161975 | 4169100 | 7125 | 4167616 | 4169100 | -1 | Twin-arginine translocation protein TatC |
| 4162559 | 4184681 | 22122 | 4162735 | 4163325 | 1 | Adenylylsulfate kinase (EC 2.7.1.25) |
| 4162559 | 4184681 | 22122 | 4164627 | 4164971 | -1 | probable glycosyl transferase |
| 4162559 | 4184681 | 22122 | 4165044 | 4166507 | 1 | hypothetical protein |
| 4162559 | 4184681 | 22122 | 4167616 | 4169100 | -1 | Twin-arginine translocation protein TatC |
| 4162559 | 4184681 | 22122 | 4169197 | 4169925 | -1 | hypothetical protein |
| 4162559 | 4184681 | 22122 | 4169942 | 4171633 | -1 | hypothetical protein |
| 4162559 | 4184681 | 22122 | 4171645 | 4172628 | -1 | SN-glycerol-3-phosphate transport ATP-binding protein UgpC (TC 3.A.1.1.3) |
| 4162559 | 4184681 | 22122 | 4172933 | 4174516 | -1 | Lipid carrier : UDP-N-acetylgalactosaminyltransferase (EC 2.4.1.-) / Alpha-1,3-N-acetylgalactosamine |
| 4162559 | 4184681 | 22122 | 4174858 | 4174974 | 1 | hypothetical protein |
| 4162559 | 4184681 | 22122 | 4175242 | 4176876 | -1 | hypothetical protein |
| 4162559 | 4184681 | 22122 | 4177566 | 4179575 | -1 | General secretion pathway protein D |
| 4162559 | 4184681 | 22122 | 4180683 | 4181594 | -1 | Transcriptional regulator, AraC family |
| 4162559 | 4184681 | 22122 | 4181739 | 4182572 | 1 | Short-chain dehydrogenase/reductase SDR |
| 4162559 | 4184681 | 22122 | 4182662 | 4183024 | -1 | FIG00955484: hypothetical protein |
| 4162559 | 4184681 | 22122 | 4183021 | 4183638 | -1 | FIG00960948: hypothetical protein |
| 4162559 | 4184681 | 22122 | 4183948 | 4184601 | 1 | FIG00958688: hypothetical protein |
| 4290214 | 4298238 | 8024 | 4289273 | 4290217 | 1 | 4-hydroxyproline epimerase (EC 5.1.1.8) |
| 4290214 | 4298238 | 8024 | 4290214 | 4291329 | 1 | D-amino-acid oxidase (EC 1.4.3.3) |
| 4290214 | 4298238 | 8024 | 4291326 | 4291562 | 1 | Hypothetical, similar to sarcosine oxidase alpha subunit, 2Fe-2S domain |
| 4290214 | 4298238 | 8024 | 4291559 | 4292812 | 1 | Putative oxidoreductase in 4-hydroxyproline catabolic gene cluster |
| 4290214 | 4298238 | 8024 | 4292809 | 4293711 | -1 | Permease of the drug/metabolite transporter (DMT) superfamily |
| 4290214 | 4298238 | 8024 | 4293811 | 4294677 | 1 | Transcriptional regulator, ArsR family |
| 4290214 | 4298238 | 8024 | 4294974 | 4295837 | -1 | putative DNA-binding protein |
| 4290214 | 4298238 | 8024 | 4295957 | 4297399 | 1 | Probable MFS transporter |
| 4290214 | 4298238 | 8024 | 4297441 | 4298238 | 1 | Transcriptional regulator, AraC family |
| 4419995 | 4427232 | 7237 | 4420183 | 4421388 | 1 | Phage integrase family domain protein |
| 4419995 | 4427232 | 7237 | 4421381 | 4422838 | 1 | Integrase |
| 4419995 | 4427232 | 7237 | 4422819 | 4424942 | 1 | miscellaneous; hypothetical/partial homology |
| 4419995 | 4427232 | 7237 | 4426758 | 4428740 | 1 | Type I restriction-modification system, DNA-methyltransferase subunit M (EC 2.1.1.72) |
| 4420183 | 4424942 | 4759 | 4420183 | 4421388 | 1 | Phage integrase family domain protein |
| 4420183 | 4424942 | 4759 | 4421381 | 4422838 | 1 | Integrase |
| 4420183 | 4424942 | 4759 | 4422819 | 4424942 | 1 | miscellaneous; hypothetical/partial homology |
| 4437997 | 4446409 | 8412 | 4437233 | 4440043 | -1 | Mobile element protein |
| 4437997 | 4446409 | 8412 | 4440176 | 4440940 | -1 | Mobile element protein |
| 4437997 | 4446409 | 8412 | 4442075 | 4442914 | -1 | Dihydropteroate synthase (EC 2.5.1.15) |
| 4437997 | 4446409 | 8412 | 4444508 | 4445140 | -1 | Streptomycin 3''-O-adenylyltransferase (EC 2.7.7.47) @ Spectinomycin 9-O-adenylyltransferase |
| 4437997 | 4446409 | 8412 | 4445392 | 4446651 | -1 | Chloramphenicol resistance protein |
| 4465850 | 4470726 | 4876 | 4465850 | 4467133 | -1 | Integrase |
| 4465850 | 4470726 | 4876 | 4467130 | 4468956 | -1 | Pyruvate/2-oxoglutarate dehydrogenase complex, dihydrolipoamide acyltransferase (E2) component, and |
| 4465850 | 4470726 | 4876 | 4469497 | 4470726 | -1 | Putative n-hydroxybenzoate hydroxylase |
| 4683660 | 4691884 | 8224 | 4683439 | 4683753 | 1 | hypothetical protein |
| 4683660 | 4691884 | 8224 | 4685813 | 4687477 | 1 | Sulfur carrier protein adenylyltransferase ThiF |
| 4683660 | 4691884 | 8224 | 4688963 | 4689142 | 1 | hypothetical protein |
| 4683660 | 4691884 | 8224 | 4691303 | 4691581 | -1 | Resolvase |
| 4838462 | 4848500 | 10038 | 4838675 | 4840180 | 1 | Hyphotheical protein |
| 4838462 | 4848500 | 10038 | 4843136 | 4843252 | 1 | hypothetical protein |
| 4838462 | 4848500 | 10038 | 4846632 | 4847138 | -1 | resolvase, putative |
| 4849740 | 4854422 | 4682 | 4850482 | 4851627 | 1 | putative transposase |
| 4849740 | 4854422 | 4682 | 4852215 | 4852742 | 1 | ATPase domain protein |
| 4850482 | 4884642 | 34160 | 4850482 | 4851627 | 1 | putative transposase |
| 4850482 | 4884642 | 34160 | 4852215 | 4852742 | 1 | ATPase domain protein |
| 4850482 | 4884642 | 34160 | 4854459 | 4854722 | 1 | Mobile element protein |
| 4850482 | 4884642 | 34160 | 4854746 | 4855597 | 1 | Mobile element protein |
| 4850482 | 4884642 | 34160 | 4858927 | 4860735 | -1 | hypothetical protein |
| 4850482 | 4884642 | 34160 | 4860735 | 4863191 | -1 | 5-methylcytosine-specific restriction related enzyme |
| 4850482 | 4884642 | 34160 | 4863191 | 4864681 | -1 | hypothetical protein |
| 4850482 | 4884642 | 34160 | 4864665 | 4868057 | -1 | DNA helicase |
| 4850482 | 4884642 | 34160 | 4868064 | 4869467 | -1 | Putative kinase protein |
| 4850482 | 4884642 | 34160 | 4869473 | 4869670 | -1 | hypothetical protein |
| 4850482 | 4884642 | 34160 | 4870165 | 4870836 | -1 | hypothetical protein |
| 4850482 | 4884642 | 34160 | 4871525 | 4877860 | -1 | helicase related protein |
| 4850482 | 4884642 | 34160 | 4877853 | 4881188 | -1 | FIG01047590: hypothetical protein |
| 4850482 | 4884642 | 34160 | 4881593 | 4883422 | -1 | predicted ATP-dependent endonuclease, OLD family |
| 4855630 | 4883411 | 27781 | 4858927 | 4860735 | -1 | hypothetical protein |
| 4855630 | 4883411 | 27781 | 4860735 | 4863191 | -1 | 5-methylcytosine-specific restriction related enzyme |
| 4855630 | 4883411 | 27781 | 4863191 | 4864681 | -1 | hypothetical protein |
| 4855630 | 4883411 | 27781 | 4864665 | 4868057 | -1 | DNA helicase |
| 4855630 | 4883411 | 27781 | 4868064 | 4869467 | -1 | Putative kinase protein |
| 4855630 | 4883411 | 27781 | 4869473 | 4869670 | -1 | hypothetical protein |
| 4855630 | 4883411 | 27781 | 4870165 | 4870836 | -1 | hypothetical protein |
| 4855630 | 4883411 | 27781 | 4871525 | 4877860 | -1 | helicase related protein |
| 4855630 | 4883411 | 27781 | 4877853 | 4881188 | -1 | FIG01047590: hypothetical protein |
| 4855630 | 4883411 | 27781 | 4881593 | 4883422 | -1 | predicted ATP-dependent endonuclease, OLD family |
| 4892972 | 4897511 | 4539 | 4891827 | 4893308 | -1 | Sigma-54 dependent DNA-binding response regulator |
| 4892972 | 4897511 | 4539 | 4895023 | 4896552 | 1 | FIG00962130: hypothetical protein |
| 5680912 | 5686037 | 5125 | 5684666 | 5684824 | 1 | hypothetical protein |
| 5680912 | 5686037 | 5125 | 5685349 | 5685573 | 1 | hypothetical protein |
| 5680912 | 5686037 | 5125 | 5685633 | 5685785 | 1 | hypothetical protein |
| 6102009 | 6108039 | 6030 | 6102303 | 6102608 | -1 | hypothetical protein |
| 6102009 | 6108039 | 6030 | 6102605 | 6103045 | -1 | hypothetical protein |
| 6102009 | 6108039 | 6030 | 6103574 | 6104809 | -1 | hypothetical protein |
| 6102009 | 6108039 | 6030 | 6104852 | 6105196 | 1 | hypothetical protein |
| 6102009 | 6108039 | 6030 | 6105168 | 6105383 | 1 | hypothetical protein |
| 6102009 | 6108039 | 6030 | 6105565 | 6106326 | 1 | Predicted Zn peptidase |
| 6102009 | 6108039 | 6030 | 6107568 | 6107969 | 1 | Putative bacteriophage-related protein |
| 6102009 | 6108039 | 6030 | 6107975 | 6109378 | 1 | Site-specific recombinase |
| 6103045 | 6132231 | 29186 | 6103574 | 6104809 | -1 | hypothetical protein |
| 6103045 | 6132231 | 29186 | 6104852 | 6105196 | 1 | hypothetical protein |
| 6103045 | 6132231 | 29186 | 6105168 | 6105383 | 1 | hypothetical protein |
| 6103045 | 6132231 | 29186 | 6105565 | 6106326 | 1 | Predicted Zn peptidase |
| 6103045 | 6132231 | 29186 | 6107568 | 6107969 | 1 | Putative bacteriophage-related protein |
| 6103045 | 6132231 | 29186 | 6107975 | 6109378 | 1 | Site-specific recombinase |
| 6103045 | 6132231 | 29186 | 6109375 | 6109839 | 1 | elements of external origin; phage-related functions and prophages |
| 6103045 | 6132231 | 29186 | 6110014 | 6110277 | 1 | elements of external origin |
| 6103045 | 6132231 | 29186 | 6110289 | 6110765 | 1 | hypothetical protein |
| 6103045 | 6132231 | 29186 | 6110765 | 6111052 | 1 | hypothetical protein |
| 6103045 | 6132231 | 29186 | 6111055 | 6111813 | 1 | Phage antirepressor protein |
| 6103045 | 6132231 | 29186 | 6111813 | 6112673 | 1 | Methyl-accepting chemotaxis protein |
| 6103045 | 6132231 | 29186 | 6112679 | 6113308 | 1 | FIG00973834: hypothetical protein |
| 6103045 | 6132231 | 29186 | 6113318 | 6113806 | 1 | FIG01111859: hypothetical protein |
| 6103045 | 6132231 | 29186 | 6113803 | 6114540 | 1 | Bucepa02001210 protein family (probably prophage associated) |
| 6103045 | 6132231 | 29186 | 6114537 | 6114764 | 1 | hypothetical protein |
| 6103045 | 6132231 | 29186 | 6114757 | 6117048 | 1 | DNA primase/helicase, phage-associated |
| 6103045 | 6132231 | 29186 | 6117297 | 6117650 | 1 | elements of external origin; phage-related functions and prophages |
| 6103045 | 6132231 | 29186 | 6117652 | 6117861 | 1 | hypothetical protein |
| 6103045 | 6132231 | 29186 | 6117854 | 6118252 | 1 | FIG00807861: hypothetical protein |
| 6103045 | 6132231 | 29186 | 6118611 | 6120026 | 1 | DNA modification methylase |
| 6103045 | 6132231 | 29186 | 6120023 | 6121285 | 1 | Adenine-specific methyltransferase (EC 2.1.1.72) |
| 6103045 | 6132231 | 29186 | 6121249 | 6121617 | -1 | Topoisomerase IA |
| 6103045 | 6132231 | 29186 | 6121715 | 6122278 | -1 | miscellaneous; unknown |
| 6103045 | 6132231 | 29186 | 6122374 | 6122580 | -1 | hypothetical protein |
| 6103045 | 6132231 | 29186 | 6122978 | 6123232 | 1 | elements of external origin; phage-related functions and prophages |
| 6103045 | 6132231 | 29186 | 6123238 | 6125190 | 1 | Phage terminase, large subunit |
| 6103045 | 6132231 | 29186 | 6125221 | 6125712 | 1 | hypothetical protein |
| 6103045 | 6132231 | 29186 | 6125712 | 6126104 | 1 | miscellaneous; unknown |
| 6103045 | 6132231 | 29186 | 6126104 | 6126325 | 1 | miscellaneous; unknown |
| 6103045 | 6132231 | 29186 | 6126417 | 6127841 | 1 | Phage portal protein |
| 6103045 | 6132231 | 29186 | 6127875 | 6129116 | 1 | Head-tail preconnector protein GP5 |
| 6103045 | 6132231 | 29186 | 6129118 | 6129495 | 1 | PROBABLE BACTERIOPHAGE-RELATED PROTEIN |
| 6103045 | 6132231 | 29186 | 6129498 | 6130502 | 1 | elements of external origin; phage-related functions and prophages |
| 6103045 | 6132231 | 29186 | 6130502 | 6130804 | 1 | FIG00808298: hypothetical protein |
| 6103045 | 6132231 | 29186 | 6130809 | 6131255 | 1 | PROBABLE SIGNAL PEPTIDE PROTEIN |
| 6103045 | 6132231 | 29186 | 6131261 | 6131482 | 1 | hypothetical protein |
| 6103045 | 6132231 | 29186 | 6131479 | 6132231 | 1 | Phage protein |
| 6130405 | 6155237 | 24832 | 6129498 | 6130502 | 1 | elements of external origin; phage-related functions and prophages |
| 6130405 | 6155237 | 24832 | 6130502 | 6130804 | 1 | FIG00808298: hypothetical protein |
| 6130405 | 6155237 | 24832 | 6130809 | 6131255 | 1 | PROBABLE SIGNAL PEPTIDE PROTEIN |
| 6130405 | 6155237 | 24832 | 6131261 | 6131482 | 1 | hypothetical protein |
| 6130405 | 6155237 | 24832 | 6131479 | 6132231 | 1 | Phage protein |
| 6130405 | 6155237 | 24832 | 6132243 | 6132641 | 1 | FIG00977876: hypothetical protein |
| 6130405 | 6155237 | 24832 | 6132659 | 6132847 | 1 | miscellaneous; unknown |
| 6130405 | 6155237 | 24832 | 6132822 | 6133466 | 1 | FIG00808222: hypothetical protein |
| 6130405 | 6155237 | 24832 | 6133468 | 6137505 | 1 | putative tail length tape measure protein |
| 6130405 | 6155237 | 24832 | 6137542 | 6137952 | 1 | FIG00808623: hypothetical protein |
| 6130405 | 6155237 | 24832 | 6137952 | 6141560 | 1 | hypothetical protein |
| 6130405 | 6155237 | 24832 | 6141608 | 6142873 | 1 | FIG00974257: hypothetical protein |
| 6130405 | 6155237 | 24832 | 6142877 | 6143962 | 1 | Conserved domain protein |
| 6130405 | 6155237 | 24832 | 6143959 | 6144354 | 1 | hypothetical protein |
| 6130405 | 6155237 | 24832 | 6144358 | 6146385 | 1 | hypothetical protein |
| 6130405 | 6155237 | 24832 | 6146378 | 6146599 | 1 | hypothetical protein |
| 6130405 | 6155237 | 24832 | 6146679 | 6146984 | 1 | PROBABLE TRANSMEMBRANE PROTEIN |
| 6130405 | 6155237 | 24832 | 6146981 | 6147208 | 1 | hypothetical protein |
| 6130405 | 6155237 | 24832 | 6147232 | 6147705 | 1 | Phage-related lysozyme (muraminidase) |
| 6130405 | 6155237 | 24832 | 6147702 | 6148202 | 1 | possible signal peptide |
| 6130405 | 6155237 | 24832 | 6149519 | 6151354 | -1 | protein of unknown function DUF262 |
| 6130405 | 6155237 | 24832 | 6151374 | 6151961 | -1 | VrlQ |
| 6130405 | 6155237 | 24832 | 6152093 | 6154108 | -1 | conserved hypothetical protein VrlP |
| 6130405 | 6155237 | 24832 | 6154105 | 6156912 | -1 | helicase (Snf2/Rad54 family) |
| 6163925 | 6175717 | 11792 | 6160201 | 6163941 | -1 | Exonuclease SbcC |
| 6163925 | 6175717 | 11792 | 6163965 | 6164426 | -1 | VrlJ |
| 6163925 | 6175717 | 11792 | 6164431 | 6165276 | -1 | COGs COG2378 |
| 6163925 | 6175717 | 11792 | 6165549 | 6169493 | 1 | DNA helicase, putative |
| 6163925 | 6175717 | 11792 | 6169490 | 6171922 | 1 | hypothetical protein |
| 6163925 | 6175717 | 11792 | 6173053 | 6174906 | 1 | ClpB protein |

**
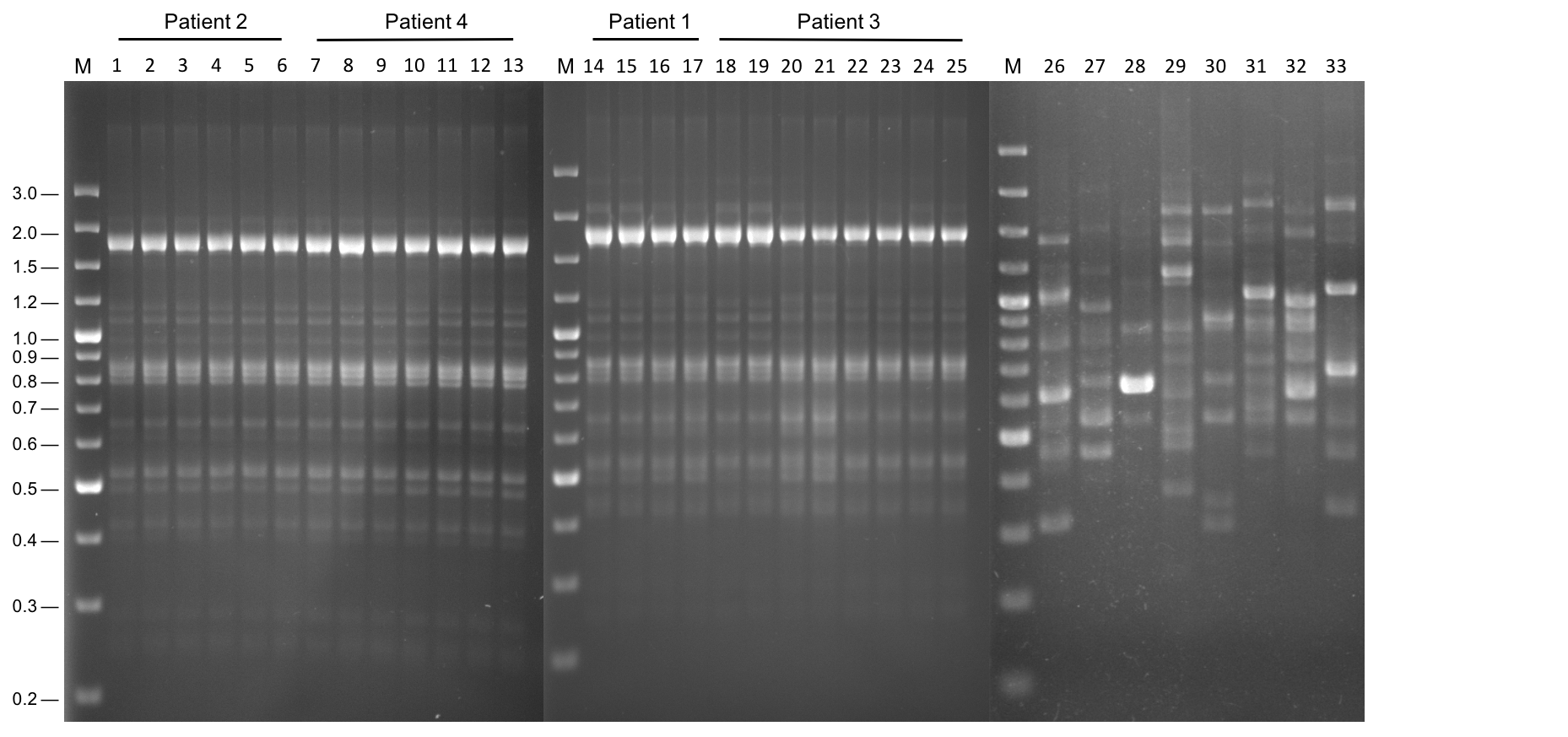
Figure S1. Random amplified polymorphic DNA (RAPD) typing of the 25 isolates and 8 other strains.** RAPD was performed for all the 25 isolates in this study to confirm their clonal linkage. We also included three laboratory strains and 5 clinical isolates from other patients in the same hospital in RAPD as control. M: marker, 1: PA_D2, 2: PA_D4, 3: PA_D6, 4: PA_D10, 5: PA_D12, 6: PA_D19, 7: PA_D16, 8: PA_D16, 9: PA_D17, 10: PA_D20, 11: PA_D23, 12: PA_D24, 13: PA_D25, 14: PA_D1, 15: PA_D3, 16: PA_D8, 17: PA_D9, 18: PA_D5, 19: PA_D7, 20: PA_D11, 21: PA_D13, 22: PA_D14, 23: PA_D15, 24: PA_D18, 25: PA_D22, 26: mPAO1, 27: PAO1, 28: PA14, 29: wk128, 30: wk173, 31: wk189, 32: wk228, 33: wk230.


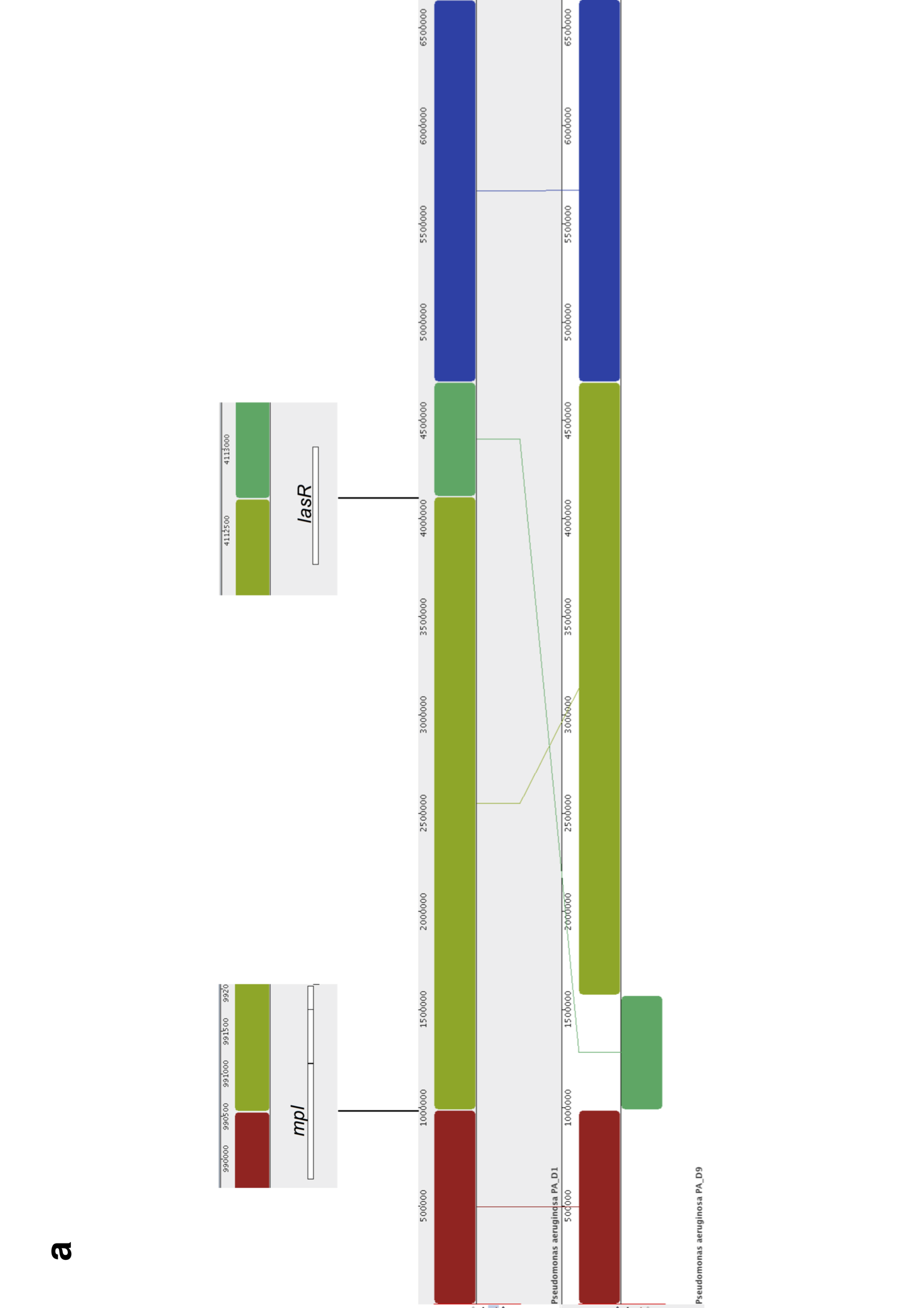
**Figure S2** **a)** **Genome alignment between PA_D1 and PA_D9.** PA_D1 and PA_D9 genomes were aligned with each other using Mauve software. Homologous segments were represented as rectangles with the same colour, which were collated with lines between the two genomes. In the PA_D9 genome, regions that are inverted compared to PA_D1 were set below to these in forward orientation. Genome rearrangement has happened between PA_D9 and PA_D1. One region has translocated to another location in PA_D9 genome. This genome rearrangement event has disrupted the *lasR* and *mpl* gene in PA_D9, as shown by zooming into the genome using Mauve software. **b) Screen for *lasR* and *mpl* deletion in all 25 isolates.** PCR was used to screen for *lasR* and *mpl* deletion. Gel images show the amplified product using primers flanking the *mpl* gene (upper panel, product size 1608 bp) and *lasR* gene (lower panel, product size 865 bp) in all 25 isolates. Both *mpl* and *lasR* were deleted in the genomes of PA_D8 and PA_D9, whereas only *lasR* was deleted from PA_D4 and PA_D19 genomes.


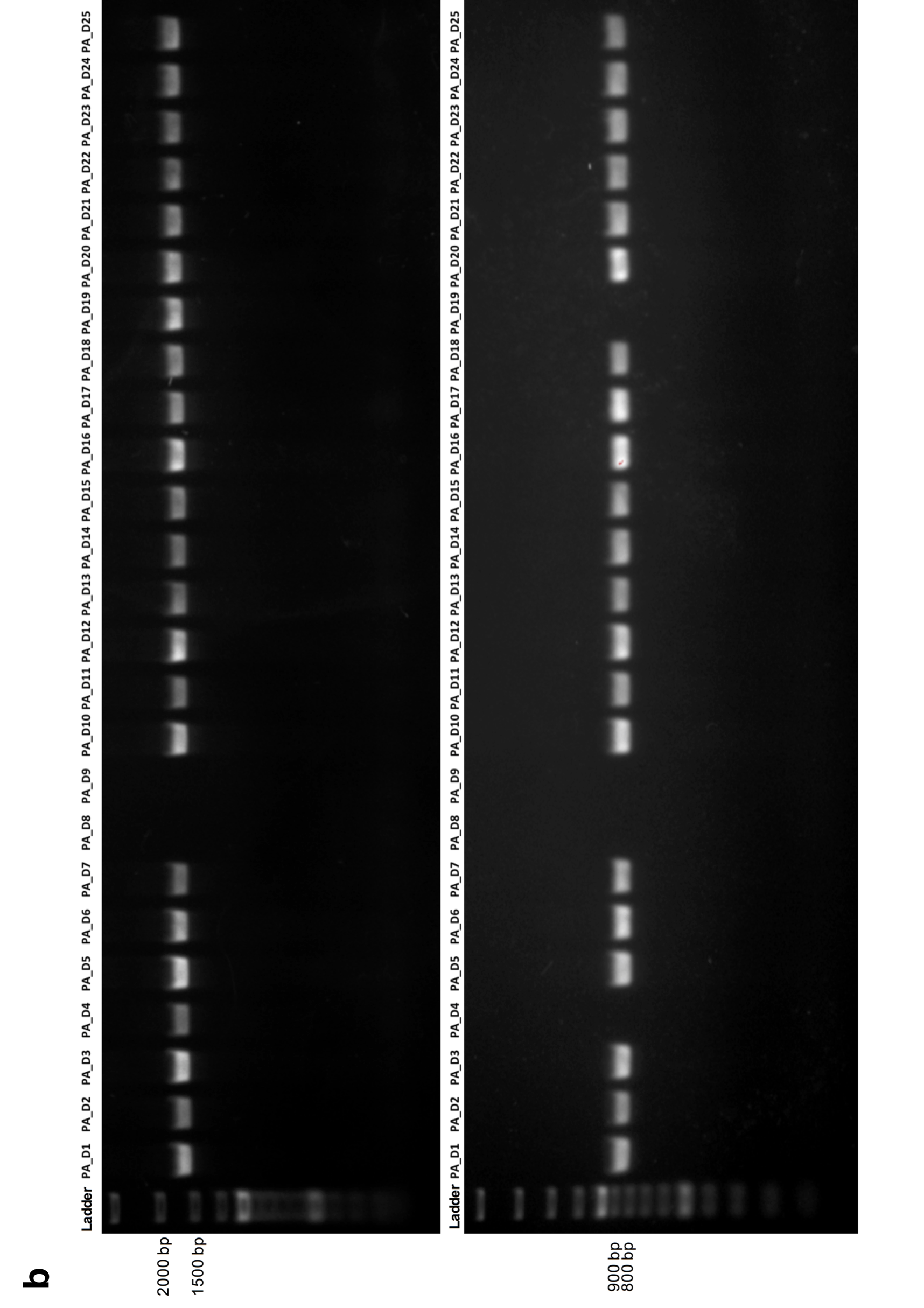

Supplement: Supplementary Figures and Tables [file rsob170029supp1.docx]
